# Extracellular Hydraulic Resistance Enhances Cell Migration

**DOI:** 10.1002/advs.202200927

**Published:** 2022-08-28

**Authors:** Debonil Maity, Kaustav Bera, Yizeng Li, Zhuoxu Ge, Qin Ni, Konstantinos Konstantopoulos, Sean X. Sun

**Affiliations:** ^1^ Department of Chemical and Biomolecular Engineering Johns Hopkins University Baltimore MD USA; ^2^ Institute of NanoBioTechnology (INBT) Johns Hopkins University Baltimore MD USA; ^3^ Department of Biomedical Engineering Binghamton University, State University of New York Binghamton NY USA; ^4^ Department of Mechanical Engineering Johns Hopkins University Baltimore MD USA; ^5^ Center for Cell Dynamics Johns Hopkins School of Medicine Johns Hopkins University Baltimore MD USA

**Keywords:** cancer, cell migration, osmotic engine model, tumor microenvironment, viscosity

## Abstract

Cells migrating in vivo encounter microenvironments with varying physical properties. One such physical variable is the fluid viscosity surrounding the cell. Increased viscosity is expected to increase the hydraulic resistance experienced by the cell and decrease cell speed. The authors demonstrate that contrary to this expected result, cells migrate faster in high viscosity media on 2‐dimensional substrates. Both actin dynamics and water dynamics driven by ion channel activity are examined. Results show that cells increase in area in high viscosity and actomyosin dynamics remain similar. Inhibiting ion channel fluxes in high viscosity media results in a large reduction in cell speed, suggesting that water flux contributes to the observed speed increase. Moreover, inhibiting actin‐dependent vesicular trafficking that transports ion channels to the cell boundary changes ion channel spatial positioning and reduces cell speed in high viscosity media. Cells also display altered Ca^2+^ activity in high viscosity media, and when cytoplasmic Ca^2+^ is sequestered, cell speed reduction and altered ion channel positioning are observed. Taken together, it is found that the cytoplasmic actin‐phase and water‐phase are coupled to drive cell migration in high viscosity media, in agreement with physical modeling that also predicts the observed cell speedup in high viscosity environments.

## Graphical abstract

The speed of a moving object in a fluid should decrease with increasing fluid viscosity. It is found that this is not true for moving mammalian cells, which speed up in high viscosity media. It is discovered that cells can sense the media viscosity. Ion channels and active water flux contribute to this observed cell speedup.

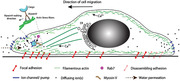

## 1 Introduction

Cells in vivo experience diverse microenvironments that can dramatically influence major biological processes such as cell migration, tissue homeostasis, repair, and disease.^[^ ^1^, ^2^, ^3^ ^]^ One such physical factor is the extracellular hydraulic resistance (eHR), which is a measure of how freely fluids can flow external to the cell. eHR is known to affect both the direction and speed of cell migration^[^ ^4^ ^]^ and depends on the geometry of the microenvironment surrounding the cell. For instance, in confining microchannels, eHR is significantly elevated and is proportional to the length of the microchannel and inversely proportional to the channel cross‐sectional area.^[^ ^5^ ^]^ In collagen‐rich extracellular matrices, eHR experienced by migrating cells can be several orders of magnitude higher than in 2D cell culture.^[^ ^5^ ^]^ Another equally important factor that influences hydraulic resistance is the viscosity of the fluid surrounding the cell.^[^ ^5^ ^]^ Indeed, eHR is directly proportional to fluid viscosity. In vivo, fluids containing significant concentrations of macromolecules are known to have elevated viscosity: synovial fluid has been demonstrated to have viscosity of ≈0.9 Pa.s.^[^ ^6^ ^]^ Human gastric mucus has a viscosity of ≈6 Pa.s.^[^ ^6^ ^]^ Airway mucus shows viscosities between 1 and 2.3 Pa.s.^[^ ^6^ ^]^ Human blood has been known to demonstrate viscosity of ≈0.005 Pa.s.^[^ ^7^ ^]^ The presence of glucose further enhances the viscosity of blood.^[^ ^8^ ^]^ In comparison, water viscosity is significantly lower, ≈0.001 Pa.s. Thus, cells are likely to encounter high viscosity fluids and physical confinement/obstacles while navigating in tissues in vivo. During cancer metastasis, cancer cells must migrate through the primary tumor site, stroma, endothelium, vascular system, and the tissue at the secondary tumor location.^[^ ^9^ ^]^ Along these steps, cancer cells are likely to encounter physical confinement, ECMs of varying densities, and fluids with high viscosity. Thus, the hydraulic resistance experienced by cancer cells can be orders of magnitude higher than what is experienced in cell culture in 2D.

Elevated eHR leads to an elevated hydraulic pressure surrounding the cell during cell migration. For the cell to translocate, the surrounding fluid must also move, which results in the development of a pressure gradient around the cell. Theoretically, this pressure gradient is directly proportional to the eHR.^[^ ^5^ ^]^ Recent data have shown that tissue cells are extraordinarily sensitive to hydraulic pressures.^[^ ^4^ ^]^ A pressure rise of a few pascals can lead to dramatic cell responses.^[^ ^4^ ^]^ Therefore, cell response to eHR is also related to cell pressure sensing. Here, we explore the effects of elevated eHR by examining 2D cell migration in high viscosity fluids. We show that, contrary to intuition which predicts that motions of objects slow down in high viscosity media due to increased fluid dynamic drag, mammalian cells such as MDA‐MB‐231 migrate faster in high viscosity fluids. The molecular mechanisms that give rise to this speed increase are explored in this work.

Cell migration is classically understood as the result of actin polymerization at the cell leading edge, cytoskeletal contraction mediated by myosin‐II, and assembly/disassembly of integrin‐dependent adhesions.^[^ ^10^ ^]^ Factors such as the mechanical properties of the underlying cell substrate, focal adhesion proteins, nuclear lamina, and LINC complexes^[^ ^11^, ^12^ ^]^ on actin‐driven cell migration have been extensively studied in 2D cell culture. However, in addition to the actin mechanism, Stroka et.al. showed that water permeation driven by ionic gradients generated by the cell can also drive migration in narrow confining channels.^[^ ^13^ ^]^ In this osmotic engine model (OEM), it was shown that Na^+^/H^+^ ion exchanger (NHE) is involved in water‐driven cell migration. NHE1 and several other ion‐channels such as sodium–potassium–chloride co‐transporter 1 (NKCC1) and sodium–potassium (NaK) pump,^[^ ^13^, ^14^, ^15^ ^]^ which have also been previously implicated in cancer, may be involved in metastasis and OEM. Li et.al., using a two‐phase model of the cytoplasm, theoretically explored the transition from actin‐driven to water‐driven mechanism of cell migration.^[^ ^16^ ^]^ The model predicted that with no change in molecular elements driving motility, cells can speed up under high hydraulic resistance.

Here, we present experimental evidence of combined actions of actin‐ and water‐driven cell migration in high extracellular viscosity environments. We demonstrate the critical role of ion channels/ion pumps in dictating motility in high viscosity fluids. Furthermore, we show that inhibition of vesicle trafficking, which impedes ion channel trafficking and spatial re‐distribution, also inhibits cell migration in high viscosity environments. Results suggest a dual role of F‐actin polymerization for motility in high viscosity conditions: it is not only involved in extending the cell leading edge but also in directing vesicular transport and positioning of ion channels that facilitate water intake. Taken together, we find that water and F‐actin contribute to the cell speed increase observed in high viscosity environments.

## 2 Results

### 2.1 Increased Media Fluid Viscosity Increases Cell Migration Speed

**Figure 1:**
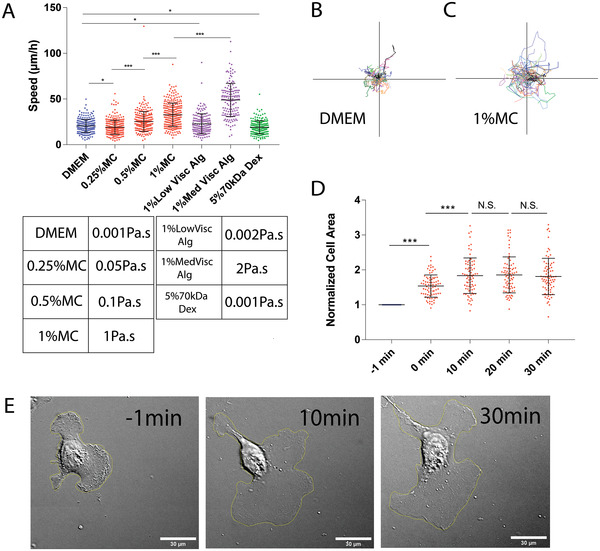
Cell migration speed and cell area increase with increasing extracellular fluid viscosity. A) Measured cell migration speed (instantaneous velocity magnitude) increases as the viscosity of the media is changed. Speeds of MDA‐MB 231 cells in DMEM (*N*=253), 0.25% MC (*N*=295), 0.5% MC (*N*=250), 1% MC (*N*=278) are shown, together with cell speeds in 1% low viscosity alginate (*N* = 167) and 1% medium viscosity alginate (*N* = 148). Cell speed is not significantly changed in media with 5% 70 kDa Dextran (*N* = 199). The table shows measured viscosity of media used. B,C) Representative cell trajectories of MDA‐MB 231 cells in DMEM and 1% MC, respectively. Cells traverse greater net distances in 1% MC media. D) Cell area, normalized with respect to the area immediately before switching to viscous media, as a function of time after the addition of 1% MC media (*N* = 79 cells). E) Representative DIC images of a single cell before (at *t* = −1 min) and after (at *t* = 10 and 30 min) media viscosity change (*N* = 79 cells). *p < 0.05, **p < 0.005, and ***p < 0.0005 across all plots. Error bars represent standard deviation of the data.

As described in the Introduction, the eHR experienced by the cell is directly proportional to the viscosity of the extracellular fluid. To investigate the effect of eHR on migrating single cells, we examined MDA‐MB‐231 cells in 2D culture in media with thickening agents such as hydroxy‐propyl‐methylcellulose (MC) and low/medium viscosity sodium alginate. With increasing % weight of MC and increasing media viscosity from 0.05 to 1 Pa.s (compared to control media where the viscosity is 0.001 Pa.s., measured using a rheometer), we observed a gradual cell speed increase (**Figure** **1**A; Video S1, Supporting Information). Here, cell speed is the magnitude of instantaneous cell velocity recorded from cell trajectories (see Experimental Section). The same trend was observed with low (0.002 Pa.s) and intermediate (2 Pa.s) viscosity media with the addition of sodium alginate (Figure 1A), implying that the cell speed increase does not depend on the chemical composition of the thickening agent.

To check that the observed speed increase was not due to media osmolarity change, we also added 5% 70 kDa Dextran, which increased the media osmolarity by 0.667 mOsm as compared to 0.116 mOsm increase generated by 1% MC. 70 kDa Dextran failed to alter both the media viscosity and the cell speed, as compared to 1% MC (Figure 1A). We also validated our observations using multiple cell lines: 3T3s, HT1080s, SUM‐159s; all showed a similar cell speed increase with increasing viscosity (Figure S1D–L, Supporting Information). Cells also displayed a significant morphological change: upon a sudden switch to high viscosity media (see Experimental Section), there is an immediate increase in cell area, up to a plateau (*t* = 30 min for MDA‐MB‐231 cells, Figure 1D,E). MDA‐MB‐231 cells also travelled larger distances (mean squared displacement, or MSD) in the same amount of time in high viscosity media (Figure 1B,C; Figure S1B,E,H,K, Supporting Information). We did not observe any significant trend with changes in cell trajectory persistence, or temporal change in angular displacement (Figure S1C,F,I,L, Supporting Information). This implies that the MSD increase is not due to cells moving more persistently in high viscosity media, compared to control media. The observed speed increase is not due to changes in cell persistence.

### 2.2 Total F‐Actin, Focal Adhesions Density, and Phosphorylated‐Myosin Light Chain (pMLC) are Unchanged While Actin Retrograde Flow Speed is Reduced in High Viscosity Media

**Figure 2:**
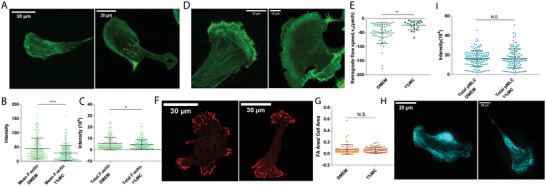
Total Filamentous‐actin (F‐Actin), Phospho‐Myosin Light Chain (pMLC), and Focal adhesion density do not change while actin retrograde flow speed reduces in high viscosity media. A) Representative fluorescence images of F‐Actin obtained from phalloidin staining, cell in DMEM (left), and cell in 1% MC (right). B,C) Comparisons of mean (intensity per unit area) and total F‐Actin for cells in DMEM (*N* = 300) and 1% MC (*N* = 250). Mean F‐Actin intensity reduces in high viscosity medium. D) Representative fluorescent images of live cells transfected with F‐Tractin in DMEM (left) and in 1% MC (right). Live cell movies are used to obtain actin retrograde flow analysis (see Experimental Section). E) Comparisons of actin retrograde flow speeds in DMEM (*N* = 44) and 1% MC (*N* = 19). F) Representative immunofluorescence images of vinculin, which are used to quantify cell focal adhesions in DMEM (left) and in 1% MC (right). G) Focal adhesion density (# per area) in cells in DMEM (*N* = 74) and 1% MC (*N* = 75). H) Representative immunofluorescence images of pMLC in cells in DMEM (left) and in 1% MC (right). I) Total intensity of pMLC in cells in DMEM (*N* = 142) and 1% MC (*N* = 124). *p < 0.05, **p < 0.005, and ***p < 0.0005 across all plots. Error bars represent standard deviation of the data.

It is known that cell migration in 2D in normal media involves the actions of F‐actin, cytoskeletal contractility, and focal adhesions. Thus, we first investigated the change in F‐actin under the influence of high viscosity media using phalloidin staining (**Figure** **2**A). We found no increase in total F‐actin, but instead a reduction in F‐actin density as measured by phalloidin fluorescence intensity per unit area (Figure 2B,C). This observation can be explained by the cell area increase after media viscosity change (Figure 1D). These findings imply that F‐actin was re‐distributed in cells exposed to high viscosity media but increased eHR did not lead to a net increase in F‐actin. Actin retrograde flow has been found to be a key element during cell migration^[^ ^17^, ^18^ ^]^ and is driven by actin polymerization and myosin contraction.^[^ ^17^ ^]^ Our analysis indicates that the actin retrograde flow speed is reduced in high viscosity media (Figure 2D,E; Video S2, Supporting Information), consistent with the faster cell speed observed in elevated eHR. Active contractile force in cells, generated mostly from myosin II acting on actin filaments, also contributes to cell migration. To check this, we stained for phosphorylated myosin light chain (pMLC) and did not observe any significant change in total pMLC (Figure 2H,I) or in the total traction stress, as measured by traction force microscopy,^[^ ^19^ ^]^ in high viscosity media (Figure S2, Supporting Information). We also stained for the focal adhesion protein vinculin and found that the number of focal adhesions per unit cell area remains the same (Figure 2F,G), implying eHR does not cause an increase in focal adhesion assembly. Taken together, our data suggest that the viscosity‐induced speed increase cannot be solely attributed to changes in the level of F‐Actin, actin retrograde flow, cell contractility, or focal adhesions.

### 2.3 Water Flux Plays a Critical Role in Cell Motility at Elevated Viscosities

**Figure 3:**
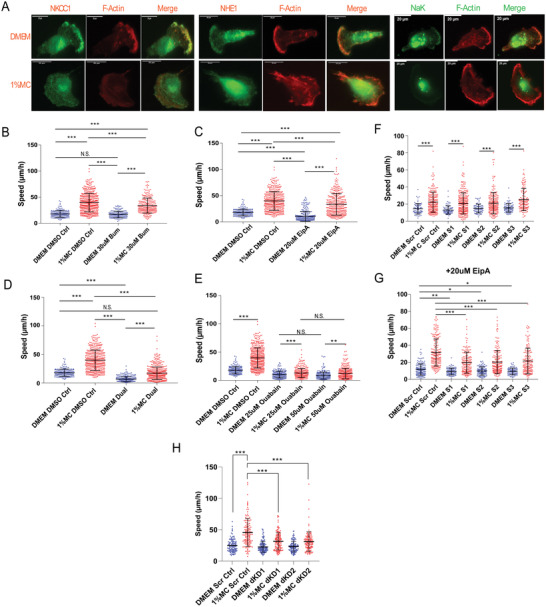
Inhibiting ion‐channel activity significantly reduces cell migration speed in high viscosity media. A) Representative immunofluorescence images of NKCC1, NHE1, NaK and F‐Actin of cells in DMEM and 1% MC. B) Cell speed upon inhibition of NKCC1 via 30 µm Bumetanide (DMEM+DMSO Ctrl *N* = 182; 1% MC DMSO Ctrl *N* = 411; DMEM 30 µm Bum *N* = 171; 1% MC 30 µm Bum *N* = 263). C) Cell speed upon inhibition of NHE1 via 20 µm EIPA (DMEM+DMSO Ctrl *N* = 182; 1% MC DMSO Ctrl *N* = 411; DMEM 20 µM EIPA *N* = 293; 1% MC 20 µm EipA *N* = 346). D) Cell speed upon dual‐ inhibition of NKCC1 and NHE1 via 30 µm Bumetanide and 20 µm EIPA (DMEM+DMSO Ctrl *N* = 182; 1% MC DMSO Ctrl *N* = 411; DMEM Dual *N* = 264; 1% MC Dual *N* = 461). E) Cell speed upon inhibition of NaK via 25 µm and 50 µm Ouabain (DMEM+DMSO Ctrl *N* = 182; 1% MC DMSO Ctrl *N* = 411; DMEM 25 µm Ouabain *N* = 202; 1% MC 25 µm Ouabain *N* = 288; DMEM 50 µm Ouabain *N* = 248; 1% MC 50 µm Ouabain *N* = 331). F) Cell speed upon NKCC1 knockdown from shRNA (DMEM Scr Ctrl *N* = 74; 1% MC Scr Ctrl *N* = 224; DMEM S1 *N* = 74; 1% MC S1 *N* = 301; DMEM S2 *N* = 71; 1% MC S2 *N* = 217; DMEM S3 *N* = 77; 1% MC S3 *N* = 133). G) Cell speed for NKCC1 knockdown cells with 20 µm EIPA (DMEM Scr Ctrl *N* = 14; 1% MC Scr Ctrl *N* = 17; DMEM S1 *N* = 13; 1% MC S1 *N* = 166; DMEM S2 *N* = 117; 1% MC S2 *N* = 188; DMEM S3 *N* = 84; 1% MC S3 *N* = 160). H) Cell Speed for NKCC1 and NHE1 double knockdown (dKD) (DMEM Scr Ctrl *N* = 107; 1% MC Scr Ctrl *N* = 126; DMEM dKD1 *N* = 125; 1% MC dKD1 *N* = 126; DMEM dKD2 *N* = 116; 1% MC dKD2 *N* = 123). *p < 0.05, **p < 0.005 and ***p < 0.0005 across all plots. Error bars represent standard deviation of the data.

It has been shown previously that transmembrane water flux can also drive cell motility.^[^ ^13^, ^16^, ^20^ ^]^ Furthermore, modeling studies revealed that both actin polymerization and water flux can contribute to the observed cell speed.^[^ ^21^ ^]^ In cells, transmembrane water flux is driven by ion concentration gradients across the cell membrane. These gradients are generated by ion channels and ion pumps. To investigate whether water permeation influences cell motility in high viscosity media, we performed immunofluoresence staining of NKCC1, NHE1, and NaK ion transporters and pumps (**Figure** **3**A). We also performed pharmacological inhibition of ion‐channel/pump NKCC1 (via Bumetanide),^[^ ^22^ ^]^ NHE1 (via EIPA),^[^ ^13^ ^]^ and NaK (via Ouabain).^[^ ^23^ ^]^ NKCC1 is a Na^+^, K^+^, and Cl^–^ co‐transporter that plays a critical role in maintaining K^+^ and Cl^–^ homeostasis. NHE1 dictates electroneutral exchange of extracellular Na^+^ and intracellular H^+^,^[^ ^24^ ^]^ and has been shown to influence cell motility in confinement.^[^ ^13^ ^]^ We observed that inhibition of either NKCC1 or NHE1 causes a small but significant reduction of cell speed in high viscosity media. However, dual inhibition of their function, either with Bumetanide + EIPA or with NKCC1 knockdown + EIPA, results in a marked reduction in cell speed (Figure 3B,C,D,F,G; Figure S3H, Supporting Information). This finding is corroborated by data generated using NKCC1 and NHE1 dual knockdown (dKD) cells using shRNA (Figure 3H), implying a synergistic role for NKCC1 and NHE1 in high viscosity media. Thus, when one ion pump is inhibited, cells are still able to generate Na^+^ flux using the other channel. It is noteworthy that in normal low viscosity media, NHE1 inhibition alone can significantly impede motility.

NaK uses a single ATP molecule to pump three Na^+^ ions out of the cell in exchange for two K^+^ ions into the cell. The pumping action of NaK can change local ion concentrations at the cell leading or trailing edge, generating water influx or efflux, driven by the osmolarity gradient. NaK has been shown to generate water fluxes.^[^ ^25^, ^26^ ^]^ Upon inhibition of NaK by Ouabain,^[^ ^23^, ^27^ ^]^ we observed a reduction in cell speed for both control and high viscosity media. However, the speed reduction in viscous media is more pronounced (Figure 3E; Video S3, Supporting Information), suggesting greater contributions of ion and water fluxes in viscous microenvironments. This conclusion is further supported by the fact that we did not observe a significant change in total F‐Actin upon treating with either Bumetanide, EIPA, or Ouabain. We only observed modest changes in the total NKCC1, NHE1, or NaK expression levels in high viscosity media (Figure S3A–C, Supporting Information). However, in DMEM, there are more ion channels at the cell leading edge (Figure S3D–F, Supporting Information), although it should be noted that more ion channels do not necessarily imply enhanced cell speed. Lastly, inhibition of ion channel activity via Ouabain, Bumetanide+ EIPA (dual inhibition), or NKCC1 and NHE1 dKD do not alter the total F‐Actin content (Figure S3G, Supporting Information) nor cell area change (Figure S3L,M,O,R, Supporting Information). As the cell area change is associated with actin dynamics, this is further indication that actin's contribution to cell speed enhancement in high viscosity is not as significant. To further establish the involvement of water flux during migration in high viscosity media, we investigated the role of aquaporins (AQPs), which enhance membrane water permeability.^[^ ^13^ ^]^ AQP5 has been found to be overexpressed in lung and breast tumor cells^[^ ^13^ ^]^ and its expression levels are higher than AQP1 and AQP3 in MDA‐MB 231 cells.^[^ ^13^ ^]^ siRNA AQP5 knockdown has been reported to inhibit confined migration.^[^ ^13^ ^]^ Upon shRNA AQP5 knockdown, we observed a reduction of cell speed in high viscosity media (Figure S3I, Supporting Information) whereas in control, we did not observe any difference in 2D.

### 2.4 Inhibition of Vesicle Trafficking Decreases Cell Speed in High Viscosity Media

Our data suggest that ion channels/pumps play a key role in regulating cell speed at elevated eHR although their total expression levels remain unaltered. Therefore, we hypothesized that intracellular vesicular trafficking might be involved in positioning these ion channels/pump and influence cell motility. Vesicular trafficking has been shown previously to influence cell migration.^[^ ^28^, ^29^ ^]^ The microtubule and actin cytoskeletons are responsible for intracellular transport of vesicles containing ion channels. One such transport process uses Myosin V motor proteins to move cargos along actin networks. A number of small Rab GTPases have been found to regulate vesicle trafficking to ensure that cargos are delivered to the correct destination.^[^ ^30^ ^]^ Myosin V has been shown to associate with cargo vesicles in a Rab‐dependent manner.^[^ ^31^ ^]^ Inhibition of Rab7 has been shown to impede migration of cancer cells such as MDA‐MB‐231.^[^ ^32^ ^]^

**Figure 4:**
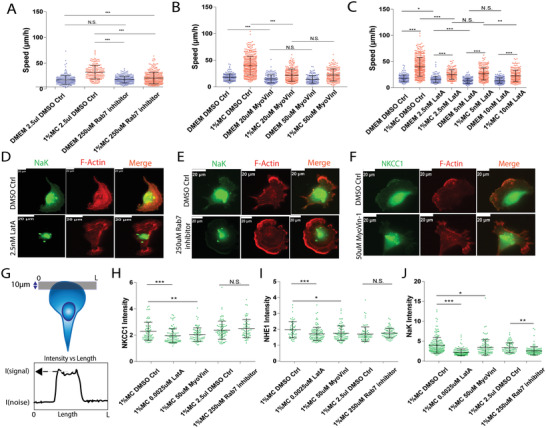
Inhibiting ion‐channel localization at the cell leading edge via perturbation of vesicle trafficking reduces cell migration speed in high viscosity media. A) Cell speed upon Rab7 inhibition via CID 1067700 (DMEM DMSO Ctrl *N* = 216; 1% MC DMSO Ctrl *N* = 205; DMEM 250 µm CID 1067700 *N*=240; 1% MC 250 µm CID 1067700 *N* = 333). B) Cell speed upon Myosin V inhibition via MyoVinI (DMEM DMSO Ctrl *N* = 182; 1% MC DMSO Ctrl *N* = 411; DMEM 20 µm MyoVinI *N* = 160; 1% MC 20 µm MyoVinI *N*=265; DMEM 50 µm MyoVinI *N* = 145; 1% MC 50 µm MyoVinI *N*= 205). C) Cell speed upon Latrunculin A treatment (DMEM DMSO Ctrl *N* = 216; 1% MC DMSO Ctrl *N* = 205; DMEM 2.5 nm LatA *N* = 174; 1% MC 2.5 nm LatA *N*=166; DMEM 5nm LatA *N* = 240; 1% MC 5 nm LatA *N*=246; DMEM 10nm LatA *N*=218; 1% MC 10 nm LatA *N* = 212). D,E,F) Representative immunofluorescence images of ion‐channels and F‐actin in cells after various treatments. G) Pictorial representation of immunofluorescence analysis for ion channels at the cell edge. A line at the leading edge is drawn and intensity within the line is recorded. For analysis, the ratio I(signal)/I(noise) is taken as leading edge intensity. H) NKCC1 leading edge intensity (1% MC DMSO Ctrl *N*=79; 1% MC 2.5 nm LatA *N* = 100; 1% MC 50 µm MyoVinI *N*=83; 1% MC 2.5 µL DMSO Ctrl *N* = 86; 1% MC 250 µm CID 1067700 *N*=66). I) NHE1 leading edge intensity (1% MC DMSO Ctrl *N*=58; 1% MC 2.5 nm LatA *N* = 111; 1% MC 50 µm MyoVinI *N*=91; 1% MC 2.5 µL DMSO Ctrl *N* = 77; 1% MC 250 µm CID 1067700 *N*=84). J) NaK leading edge intensity (1% MC DMSO Ctrl *N* = 183; 1% MC 2.5 nm LatA *N* = 136; 1% MC 50 µm MyoVinI *N* = 104; 1% MC 2.5 µL DMSO Ctrl *N* = 85; 1% MC 250 µm CID 1067700 *N* = 114). *p < 0.05, **p < 0.005, and ***p < 0.0005 across all plots. Error bars represent standard deviation of the data.

To test our hypothesis, we perturbed the trafficking of ion channels such as NHE1, NaK, and NKCC1 via Rab7 inhibition (via CID 1067700),^[^ ^33^ ^]^ Myosin‐V inhibition (via MyoVinI),^[^ ^34^ ^]^ or actin polymerization inhibition using very low concentrations (2.5–10 nm) of Latrunculin A (LatA). We observed that all these pharmacological interventions led to a significant reduction in cell speed in high viscosity media as compared to control (**Figure** **4**A–C; Video S4, Supporting Information). For the case of Myosin V inhibition, the gap between cell speeds in DMEM and high viscosity media under MyoVinI treatment is similar as observed for the case of Rab7 inhibition or LatA. We also performed immunofluorescence staining and observed that indeed LatA affects the localization of NHE1, NKCC1, and NaK at the leading edge of the cell. Rab7 inhibition affects only the localization of NaK with no effect on actin‐dependent cell area change in high viscosity media (Figure S4E, Supporting Information). Myosin‐V inhibition affects NKCC1, NHE1, and to a small extent NaK localization at the leading edge of the cell (Figure 4D–J), but it is not as dramatic as LatA or Rab7 inhibition. Our data showed that even without a change in the total ion channel content and/or total F‐Actin content (Figure S4A–D, Supporting Information), ion channel localization at the leading edge can be significantly altered by perturbing vesicular trafficking. These observations imply that F‐actin can not only generate cell protrusions and cell movement but also influence motility through the trafficking and localization of the pertinent ion‐channels/pumps at the cell leading edge.

If ion and water fluxes are prominent during cell migration in high hydraulic resistance conditions, then the trafficking of ion channels and pumps would also influence cell motility in 3D collagen gels. The results of inhibiting NKCC1, NHE1, NaK, and Myosin‐V show that this is indeed the case (Figure S6A, Supporting Information). However, the presence of collagen will also influence focal adhesion formation and other factors that influence motility. A thorough analysis of this condition will require more extensive study and is beyond the scope of the current work.

### 2.5 Calcium Dynamics Responds to Changes in Media Viscosity and Influences Motility

**Figure 5:**
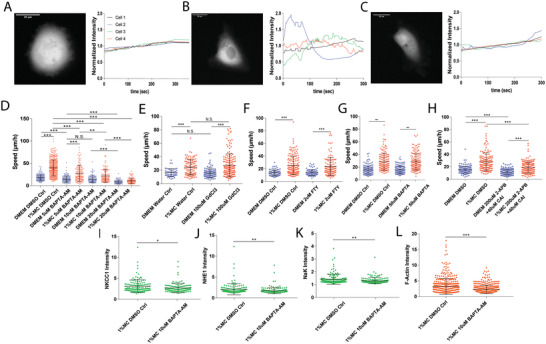
Calcium (Ca^2+^) dynamics affect cell migration speed by regulating ion‐channel and F‐actin localization at the cell leading edge. Intracellular Ca^2+^ levels (measured as fluorescence signal from GCamp6m transfected live cells) in different media conditions. Representative GCamp6m fluorescent images in A) DMEM (immediately after addition), B) 1% MC (immediately after addition), and C) 1% MC (long term >3 h incubation). D) Cell speed under various levels of Ca^2+^ chelation via BAPTA‐AM (DMEM DMSO Ctrl *N*=182; 1% MC DMSO Ctrl *N* = 411; DMEM 5 µm BAPTA‐AM *N*=135; 1% MC 5 µm BAPTA‐AM *N*=144; DMEM 10 µm BAPTA‐AM *N* = 117; 1% MC 10 µm BAPTA‐AM *N* = 137; DMEM 20 µm BAPTA‐AM *N*=86; 1% MC 20 µm BAPTA‐AM *N* = 127). E) Cell speed upon non‐specific inhibition of mechano‐sensitive channels via GdCl_3_ (DMEM Water Ctrl *N* = 45; 1% MC Water Ctrl *N* = 102; DMEM 100 µm GdCl_3_ *N*=96; 1% MC 100 µm GdCl_3_ *N* = 157). F) Cell speed upon inhibition of TRPM7 via FTY (DMEM DMSO Ctrl N=77; 1% MC DMSO Ctrl *N* = 154; DMEM 2 µm FTY *N* = 83; 1% MC 2 µm FTY *N* = 120). G) Cell speed upon treatment with extracellular Ca^2+^ chelator BAPTA (DMEM DMSO Ctrl *N* = 162; 1% MC DMSO Ctrl *N*=152; DMEM 50 µm BAPTA *N* = 146; 1% MC 50 µm BAPTA *N* = 144). H) Cell speed upon treatment with 2‐APB and CAI (DMEM DMSO Ctrl *N* = 145; 1% MC DMSO Ctrl *N* = 149; DMEM 200 µm 2‐APB + 40 µm CAI *N*=169; 1% MC 200 µm 2‐APB + 40 µm CAI *N* = 178). Leading edge intensities of ion‐channels and F‐actin are reduced under BAPTA‐AM treatment as compared to DMSO Control: I) Leading edge intensities of NKCC1 (1% MC DMSO Ctrl *N* = 132; 1% MC 10 µm BAPTA‐AM *N* = 115), J) Leading edge intensities of NHE1 (1% MC DMSO Ctrl *N* = 132; 1% MC 10 µm BAPTA‐AM *N* = 171), K) Leading edge intensities of NaK (1% MC DMSO Ctrl *N* = 133; 1% MC 10 µm BAPTA‐AM *N* = 144), and L) Leading edge intensities of F‐actin (1% MC DMSO Ctrl *N* = 397; 1% MC 10 µm BAPTA‐AM *N* = 430). *p < 0.05, **p < 0.005 and ***p < 0.0005 across all plots. Error bars represent standard deviation of the data.

Calcium (Ca^2+^) signaling has been found to be a key component during spontaneous cell polarization and has been shown to coordinate directional cell migration.^[^ ^35^ ^]^ Cell sensing of confinement‐induced eHR is Ca^2+^ dependent and is mediated by the mechano‐sensitive TRPM7 Ca^2+^ channel.^[^ ^4^ ^]^ Similarly, mechano‐sensitive Ca^2+^ channel TRPV4 has been shown to be involved in sensing mechanical compression.^[^ ^36^ ^]^ Ca^2+^ also has been shown to be involved in vesicle fusion to cell membrane, stabilizing transport vesicle coats, and hence, dictating vesicle trafficking.^[^ ^37^ ^]^ Moreover, as Ca^2+^ also regulates fluxes of many ion channels and pumps, ion channels/pumps activity is also expected to depend on calcium. Therefore, we examined the role of Ca^2+^ in cell motility in high viscosity media by transiently transfecting cells with GCamp6M, a fluorescent live cell Ca^2+^ reporter.^[^ ^38^ ^]^ We observed that at short times (minutes after contact with high viscosity media), cells showed large fluctuations in cytoplasmic Ca^2+^ levels, which ultimately stabilized after 30 min (**Figure** **5**A–C; Video S6, Supporting Information). Fast Fourier transform (FFT)^[^ ^39^ ^]^ of these signals yielded high frequency Ca^2+^ oscillations immediately after exposure to high viscosity media, while lower frequency oscillations are observed in control media and long times after media change (Figure S5A,B, Supporting Information). Interestingly, high viscosity media resulted in elevated levels of intracellular Ca^2+^ content, both at short and long times, as compared to control media (Figure S5C–F, Supporting Information).

To perturb Ca^2+^ signaling in moving cells, we used an intracellular Ca^2+^ chelator, BAPTA‐AM, and observed a significant reduction in cell speed in high viscosity media (Figure 5D). Furthermore, BAPTA‐AM caused a decrease in the leading edge localization of ion channels such as NKCC1, NHE1, and NaK (Figures 5J–L, respectively) at the leading edges of cells in high viscosity media and also reduced the local F‐Actin (Figure 5M), while the total ion channel and F‐Actin content remained the same (Figure S5G–K, Supporting Information). However, non‐specific inhibition of TRP channels using GdCl_3_ caused no significant reduction in speed in high viscosity media (Figure 5E) and TRPM7 inhibition did not affect cell motility in high viscosity media (Figure 5F). To further examine the role of intracellular Ca^2+^, we used 2‐APB (which blocks inositol 1,4,5‐trisphosphate receptor) and CAI (carboxy‐amidotriazole, which inhibits store operated Ca^2+^ channels). Upon using 2‐APB+CAI, we observed a reduction in cell speed, and significantly, more reduction in cell speed in high viscous media as compared to the normal media (Figure 5H; Video S5, Supporting Information). This observed cell speed reduction is consistent with our BAPTA‐AM experiment. Upon perturbation of extracellular Ca^2+^ via BAPTA, we did not observe any speed reduction in high viscosity media (Figure 5G), implying that perturbation of extracellular Ca^2+^ did not affect motility in high viscosity media. However, sequestering intracellular Ca^2+^ influenced the positioning of NKCC1, NHE1, and NaK at the cell leading/trailing edges (Figure 5I–K) and therefore reduced cell speed in high viscosity media.

### 2.6 Mathematical Modeling Can Explain the Observed Speed Increase in High Viscosity Media

**Figure 6:**
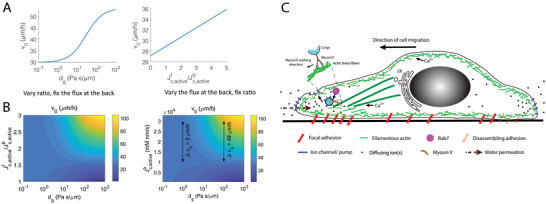
Mathematical modeling can explain cell speedup in high viscosity media. A) Model prediction of water‐driven cell migration velocity as a function of the coefficient of external hydraulic resistance (left panel) and the ratio of active ion flux at the leading and trailing edges of the cell (right panel). B) Model predictions of the water‐driven cell velocity as a function of active ion flux and the coefficient of external hydraulic resistance. Left panel: active ion flux at the back of the cell is fixed but the ratio of front‐to‐back ion flux is varied. The model predicts that the cell velocity increases with the flux ratio and the external hydraulic resistance. Right panel: the front‐to‐back active ion flux ratio is fixed while varying the ion flux at the back. This is to model the case when some ion transporters and pumps are inhibited in the experiment. The inhibition will reduce the ion flux across the cell boundary while the ratio may remain the same. The model predicts that the cell velocity reduction is more prominent at high hydraulic resistance when the overall ion flux is reduced, that is, velocity decrease by 2 µm h^−1^ when *d*_g_ = 1 Pa s µm^−1^ versus velocity decrease by 49 µm h^−1^ when *d*_g_ = 10^2^ Pa s µm^−1^. C) Schematic representation of cell motility mechanism in high viscosity conditions. Water flux‐driven mode of cell migration is facilitated by ion‐channel localization at the cell leading edge. Actin tracks, Myosin V, and Rab7 facilitate trafficking of ion‐channels to the cell leading edge. Ca^2+^ influences ion‐channel localization at the cell leading edge by modulating actin polymerization and vesicle trafficking. The direction of water influx at the cell leading edge is opposite to the direction of cell migration. Increased media viscosity and hydraulic resistance alter cell ion channel polarization and increase cell speed.

Our observed cell speed increase in higher viscosity media can be explained using a two‐phase mathematical model of the migrating cell^[^ ^16^, ^21^ ^]^ (**Figure** **6**A; see details in Supporting Information). Fundamentally, from mass conservation alone, cell boundaries can advance from both actin polymerization and water influx from the exterior into the cell.^[^ ^20^ ^]^ When there is no water flux, the actin retrograde flow velocity is determined by the rate of actin polymerization and myosin contraction. When there is water influx, for the same rate of actin polymerization (Figure 2B,C), our model predicts that the actin retrograde flow speed is reduced. This is consistent with our experimental observations (Figure 2E). Our mathematical model also predicts that increased water flux also increases cell speed, and the speed increase is elevated in high viscosity (or high hydraulic resistance) conditions (Supporting Information). Our experiments show that in addition to this physical mechanism of cell speed enhancement, cells can sense the external hydraulic resistance and as a result, ion channel/pump distribution is altered in high viscosity media. Consistent with these observations (Figure 4G–I), our model predicts that increased polarization of ion channels in high viscosity media leads to increased cell speed and explains why ion channel inhibition has a greater effect in high viscosity media (Figure 6A,B). Modeling also predicts that cell adhesion frictional force is an important factor in determining cell speed.^[^ ^16^, ^21^ ^]^

## 3 Discussion and Conclusion

By measuring the speed of cell migration in 2D in media of different viscosity, we discovered that cells can migrate faster in media with higher viscosity. This result is unexpected as most moving objects slow down when the viscosity or resistance increases. While hydraulic resistance for slow moving mammalian cells is generally negligible on 2D surfaces, MDA‐MD‐231 cells are able to sense media viscosity change and increase cell adhesion area in high viscosity media. When the media viscosity is increased by 1000‐fold, the increased hydraulic resistance is significant, especially with increased shear stress at the cell surface. Our investigations yielded a potential mechanism that can explain the cell speed increase under high eHR. We found the cell speed in high viscosity media to be driven by both F‐actin and water flux. As water flux is primarily driven by ion fluxes across the cell surface, we investigated the role of ion‐channels such as NKCC1 and NHE1, and ion pump, such as, NaK, in dictating motility in high viscosity media. We found that concurrent inhibition of NKCC1 and NHE1 markedly decreases cell speed in higher viscosity. By impeding (using LatA as well as myosin V and Rab7 inhibition) cell vesicular trafficking of these ion channels/pumps to the cell leading edge, we found that cell speed decreased significantly in high viscosity media (Figure 6). Specifically, we observed that inhibition of actin polymerization via LatA affects the localization of all ion channels, namely, NKCC1, NHE1 and NaK, while MyosinV inhibition, to a much lesser extent, affects NKCC1 and NHE1. Rab7 was found to diminish the leading‐edge localization of NaK. This result implies that actin polymerization can not only affect actin mediated cell motility but also influence water permeation mode of motility by establishing ion channel localization at the cell boundaries. However, the cell speed slow down under LatA treatment could be explained by direct interference of actin‐generated protrusions. From the MyosinV inhibition and Rab7 inhibition data, we showed that there does not need to be a dramatic change in total F‐actin content to bring about a change in ion‐channel distribution at the cell leading edge. Moreover, Ca^2+^ dynamics are involved in regulating motility in high viscosity. We found Ca^2+^ plays a key role in ion channel positioning (Figure 6). Ca^2+^ is involved in regulating ion channel trafficking and function,^[^ ^40^ ^]^ as well as in a large number of intracellular processes. Furthermore, local regulation of contractile forces has been shown to depend on global Ca^2+^,^[^ ^41^ ^]^ and local Ca^2+^ sparks allow spatial segregation of mechano‐transduction events.^[^ ^42^ ^]^ In our experiments, we found no change in the total pMLC content (in addition to focal adhesion density and traction stress) between the cases of normal and high viscosity media, implying that pMLC (just like F‐Actin) is redistributed all over the cell and there is no change in contractile forces involved. In‐depth roles of contractility and focal adhesions in influencing cell motility under high viscosity conditions will require further investigation.

We observed that TRPM7 is not involved in changing cell speed when exposed to high viscosity media, but it is involved in sensing hydraulic resistance in confinement,^[^ ^4^ ^]^ implying cells have different mechanisms in sensing and adapting to viscosity and geometry‐mediated hydraulic resistance. We also showed that the influx of Ca^2+^ through TRP channels did not affect motility and sequestering extracellular Ca^2+^ does not affect motility in high viscosity media. It is only the intracellular Ca^2+^ levels that are relevant in this 2D migration scenario.

Our motility experiments were performed with glass substrates. When the substrate was coated with 20 µg mL^−1^ of collagen, we also observed a cell speed increase in high viscosity media. However, this speed increase was not as dramatic as the case of no coating (Figure S6B, Supporting Information). With collagen coating, we found that cells contain elevated levels of F‐actin (Figure S6C,D, Supporting Information), perhaps implying that cell speed is more F‐Actin driven with collagen substrates. This could follow from that fact that integrin engagement with collagen can result in RhoA activation,^[^ ^43^ ^]^ which can result in a more actin driven motility. Moreover, experiments for cells in 3D collagen matrices also showed a strong influence of ion channels on cell speed. Indeed, the effective hydraulic resistance experienced by cells is significantly higher in 3D matrices.^[^ ^5^ ^]^ Therefore, the water‐driven mechanism of cell migration may be more prominent in 3D matrices. More studies are needed to understand how collagen ECM in 3D matrices can change cell motility mechanisms.

Another key feature that might influence cell motility is the cell endocytic cycle, which allows the cell to take up external fluid, leading to forward cell movement. We also measured the degree of cell endocytosis using high molecular weight fluorescent dye. Results show that during the time of cell speed data collection, there is no observable uptake of external media by cell endocytosis (Figure S6E, Supporting Information). We have also observed that EIPA^[^ ^44^ ^]^ inhibition of NHE1 did not cause a cell speed reduction in high viscosity media. As EIPA is involved in regulating macropinocytosis,^[^ ^44^ ^]^ we conclude that the observed cell speed increase in high viscosity media is not due to elevated endocytosis. In other situations, amoeboid cells in suspension exhibit increased endocytosis at the back and membrane trafficking from front to back.^[^ ^45^ ^]^ The endocytic cycle can impact cell polarization, cell area changes, and membrane flow,^[^ ^46^ ^]^ and therefore, is also as important as the cytoskeleton and ion channels in controlling cell motility. A possible role of the endocytic cycle in cell motility in high viscosity media is an important topic of future investigation.

In conclusion, our results show that media fluid viscosity can significantly impact cell migration dynamics. The observed cell migration speed in all environments is a combination of F‐actin polymerization and water/ion flux across the cell membrane. F‐actin serves a duel role of generating cell protrusions as well as directing vesicular trafficking that positions ion channels on the cell boundary (Figure 6C). A two‐phase model of cell migration can quantitatively explain cell speed in different conditions. Cells are able to sense media viscosity change, possibly through a Ca^2+^‐dependent mechanism, which also directs ion channel trafficking. The active sensing of media viscosity and hydraulic resistance is likely related to cell hydraulic pressure sensing, and ultimately, changes cell migration dynamics and cell speed.

## 4 Experimental Section

### Cell Culture

MDA‐MB 231, HT1080, 3T3, and Sum 159 cells were kind gifts from the lab of Prof. Konstantinos Konstantopoulos. All cells were cultured in DMEM (ThermoFisher 11995065), 10% (by volume) FBS (Sigma–Aldrich) and 1% (by volume) penicillin/streptomycin (Gibco) at 37 °C and 5%CO_2_.

### Immunostaining

For immunostaining, the cells were treated with pertinent drugs or no drugs. After treatment times, they were fixed using Paraformaldehyde Solution (4% in PBS, Thermo Scientific J19943K2) for 15 min at room temperature (RT). After fixation, cells were washed with PBS, three times for a duration of 5 min each. They were then permeabilized using 0.5% (by volume) Triton‐X (Sigma–Aldrich X100) in PBS for 10 min at RT. After permeabilization, the cells were washed with PBS in three steps. Then, the cells were subjected to blocking agent 2% (by volume) Bovine Serum Albumin (BSA) (Sigma–Aldrich A7906) in PBS for 30 min at RT. Then, the steps of incubation with primary/secondary antibodies in 1% BSA solution were carried out for a period of 1 h at RT each. The incubation with primary and/or secondary antibodies was followed by the three step washing with PBS. For F‐Actin staining, Alexa Fluor 647 Phalloidin (Thermo Fisher A22287) was used in 1:100 volumetric‐ ratio in 1% BSA. For Vinculin staining, Vinculin Monoclonal Antibody (7F9), Alexa Fluor 488 (Thermo Fisher 53‐9777‐82) in 1:25 volumetric ratio in 1% BSA was used. For staining of pMLC, 1:2000 Primary: Anti‐Myosin light chain (phospho S20) antibody (Abcam ab2480) and 1:250 Secondary: Goat Anti‐Rabbit IgG H&L (Alexa Fluor 488) (Abcam ab150077) volumetric ratio in 1% BSA were used. For NKCC1 staining, 1:100 Primary NKCC1 (D13A9) Rabbit mAb (Cell Signaling Technology 8351) and 1:100 Secondary Alexa Fluor 488 AffiniPure Goat Anti‐Rabbit IgG (H+L) (Jackson Immuno 111‐545‐003) were used. For NHE1 staining, 1:100 Primary (B‐12) (Santa Cruz sc‐515950) and 1:100 Secondary Goat anti‐Mouse IgG (H+L) Cross‐Adsorbed Secondary Antibody, Alexa Fluor 488 (Thermo Fisher A‐11001) were used. For NaK staining, 1:100 Primary Anti‐Na+/K+ ATPase *α*‐1 Antibody, clone (Sigma–Aldrich 05–369) and 1:100 Secondary Anti‐mouse IgG (H+L) (Cell Signaling Technology 4409S) were used. All were in volumetric ratio in 1%BSA.

### Cell Speed Measurements, Fluorescence Analysis

Cell speeds measurements were taken using DIC (5 h, 10 min intervals) and fluorescence was imaged using Epifluorescence in a Zeiss LSM 800 system. For cell speeds, a 10× air objective was used. For immunostaining images, 40× objective was used. For cell speed measurements, 20 000 cells were seeded in 24‐well glass bottom plates and imaged within 10–12 h of seeding. Cell speeds were quantified by identifying and following the cell center by hand. Fluorescent images were hand traced. For the analysis of immunostaining, the mean local background noise was subtracted from the image intensity before quantifi. For total distance and persistence calculations, the location of the cells obtained from 10× DIC imaging was utilized. For focal adhesion images, 63× objective and confocal in a Zeiss LSM 800 system were used. For focal adhesion area to cell area ratio, image thresholding was used to segregate focal adhesions. Then, total area of focal adhesions was obtained calculating the number of non‐zero pixels as obtained from thresholding. Cell area was calculated by hand tracing. MATLAB and ImageJ were used for all image analysis.

### Retrograde Flow Measurements

For retrograde flow measurements, the cells were transiently transfected using pEGFP‐C1 F‐tractin‐EGFP which was a gift from Dyche Mullins Lab (Addgene plasmid # 58473). After ≈20 h, the cells were incubated with fresh control media or the high viscosity media and then imaged using 63X Confocal (3 min, 3 s intervals). Particle image velocimetry (PIVLab) was used for the analysis.

### Calcium Imaging

For calcium imaging, the cells were transiently transfected with pGP‐CMV‐GCaMP6m which was a gift from Douglas Kim (Addgene plasmid # 40754). After ≈20 h, the cells were incubated with fresh control media or the high viscosity media and then imaged using 40× Epifluorescence (5 min, 1 s intervals). After fast Fourier transform of the calcium signals, power versus frequency were plotted normalized relative to the maximum power (global maxima of the power versus frequency). Then, the spectrum was divided into several bins, namely, Bin 1: 0–100mHz, Bin 2: 100–200mHz, Bin 3: 200–300mHz, Bin 4: 300–400mHz, and Bin 5: 400–500mHz. For mean relative peak power, the average of the powers of the peaks (points of local maxima) in a specific bin was taken, and for maximum relative peak power, the maximum power in a bin was taken and it was divided by the total number of peaks in that specific bin.

### Traction Force Microscopy

For 2D substrate preparation, the protocol as described in a previous article was used.^[^ ^19^ ^]^ After the substrate preparation, it was coated with 20 ug mL of rat tail type I collagen (Corning) and incubated in 37 °C for 1h. After gently taking out the collagen solution, the cells were seeded over a duration of ≈20 h to allow them enough time to adhere and spread out. For performing traction force experiments, a 40× objective was used and trypsinization was used to make the cells detach. The samples were imaged before trypsinization and >30 min after trypsinization. The bead displacements were recorded and the ensuing images were analyzed as described in a previous article.^[^ ^19^ ^]^

### Viscosity Media Preparations

For preparation of hydroxyl–propyl–methylcellulose, 0.25%, 0.5%, and 1% (weight/volume) of Methocel J75MS (Dow) were used in cell culture media. For 1% (weight/volume in cell culture media) low viscosity and media viscosity sodium alginate, Alginic acid sodium salt from brown algae (Sigma–Aldrich Low‐ A1112 and Medium A2033 viscosity respectively) was used. For 5% (weight/volume in cell culture media) Dextran solution, Dextran from Leuconostoc spp. (Sigma–Aldrich 31390) was used. Immediately after mixing the solutes, the solution was subjected to a constant rotation at 10 rpm at RT over a period of ≈1–2days in order to achieve a uniform mixture. The viscosity values were measured using a rheometer or were provided by the manufacturer.

### Inhibition Experiments

Latrunculin A (Sigma–Aldrich), 5‐(*N*‐Ethyl‐*N*‐isopropyl) amiloride (EIPA, Sigma–Aldrich), Ouabain (Sigma–Aldrich), Bumetanide (Ro 10–6338, Santa Cruz Biotechnology), Rab7 inhibitior CID 1067700 (Sigma–Aldrich), MyoVin‐1 (Sigma–Aldrich), BAPTA (Sigma–Aldrich), BAPTA‐AM (Sigma–Aldrich), FTY720, 2‐APB and CAI (Tocris Biosciences), Calpain inhibitor I (Millipore Sigma) in dimethylsulfoxide and Gadolinium (III) Chloride (Sigma–Aldrich) in water were used. For all inhibition experiments, cells were incubated in cell culture media with drugs for a duration of 1 h (except Ouabain ≈2 h) and then, high viscosity media was added. Cells were then imaged over a span of 5 h for speed measurements or were incubated over a span of ≈4 h for immunostaining.

### Collagen Gel Preparation and 3D Cell Speed Acquisition

For collagen gelation, the protocol described in a previous publication was used.^[^ ^10^ ^]^ Cells in 1:1 (v/v) ratio of cell culture media and reconstitution buffer were mixed with the appropriate volume of rat‐tail type I collagen (Corning) to obtain a final collagen I concentration of 1 mg mL^−1^. A pertinent calculated amount of 1 m NaOH was quickly added. Cells were allowed to spread out over a duration of ≈6–8 h and then, pharmacological inhibition experiments were performed. X–Y displacement of the cells were acquired using 20× DIC (7 h, 10 min intervals).

### Cloning, Lentivirus Preparation, and Transduction

To generate MDA‐MB‐231 cells with stable knock down of NKCC1, pLKO.1 puro (plasmid no. 8453; Addgene, Cambride, MA; a gift from B. Weinberg) backbone was used. Non‐specific scramble sequence (5’‐GCACTACCAGAGCTAACTCAGATAGTACT‐3’), shNKCC1 sequence‐1 (5’‐ACCAAATTTCATCCATATATC‐3’), shNKCC1 sequence‐2 (5’‐ GCCACTCTTTCTTCAGCATTA‐3’), and shNKCC1 sequence‐3 (5’‐ GCCACTCTTTCTTCAGCATTA‐3’) were subcloned into the backbone. For producing lentivirus 293T/17 cells were co‐transfected with psPAX2, pMD2.G, and the lentiviral plasmid. 48 h after transfection, lentivirus was harvested and concentrated using centrifugation. Wild type MDA‐MB231 cells at 60–80% confluency were incubated for 24 h with 100× virus suspension and 8 µg mL^−1^ of Polybrene Transfection Reagent (Millipore Sigma). To maintain stable knock down, virus transduced cells were grown in media containing 0.5 µg mL^−1^ Puromycin (Gibco). AQP5 knock‐down cell lines were a kind gift from the lab of Prof. Konstantinos Konstantopoulos.

To generate MDA‐MB‐231 cells with stable knock down of NKCC1 and NHE1, non‐specific scramble sequence (5’‐GCACTACCAGAGCTAACTCAGATAGTACT‐3’), shNKCC1 sequence‐1 (5’‐ACCAAATTTCATCCATATATC‐3’), shNKCC1 sequence‐2 (5’‐GCCACTCTTTCTTCAGCATTA‐3’), shNKCC1 sequence‐3 (5’‐GCCACTCTTTCTTCAGCATTA‐3’), shNHE1 sequence‐2 (5’‐ GACAAGCTCAACCGGTTTAAT ‐3’), and shNHE1 sequence‐7 (5’‐ CCAATCTTAGTTTCTAACCAA ‐3’) were subcloned into the pLKO.1 puro (plasmid no. 8453; Addgene, Cambride, MA; a gift from B.Weinberg) backbone. For producing lentivirus 293T/17 cells were co‐transfected with psPAX2, pMD2.G, and the lentiviral plasmid. 48 h after transfection, lentivirus was harvested and concentrated using centrifugation. Wild type MDA‐MB‐231 cells at 60–80% confluency were incubated for 24 h with 100× virus suspension and 8 µg mL^−1^ of Polybrene Transfection Reagent (Millipore Sigma). To maintain stable knock down, virus transduced cells were grown in media containing 0.5 µg mL^−1^ Puromycin (Gibco).

### Quantitative PCR

Scramble control and shNKCC1 cells were grown to 95% confluency and their total RNA was isolated using Direct‐zol RNA isolation kit (Zymo Research) according to manufacturer instructions. Reverse Transcription and Quantitative PCR were performed using standard techniques using the following primer set for NKCC1: F‐(5’‐CCTCTACACAAGCCCTGACTTAC‐3’) and R‐(5’‐CGTGAGTTTGGAGCACCTGTCA ‐3’); NHE1: F‐(5’‐ ACCTGGTTCATCAACAAGTTCCG ‐3’) and R‐(5’‐ TTCACAGCCAACAGGTCTACCA ‐3’).

### Western Blotting

Western blots were performed using NuPage 4% to 12% bistris gels and the following antibodies: anti NKCC1 (raised in rabbit; clone D13A9; Cell Signaling Technology 8351), and anti NHE1 (raised in mouse; clone 54; Santa Cruz Biotechnology; sc‐136239) with glyceraldehyde‐3‐phosphate dehydrogenase (rabbit, clone 14C10, Cell Signaling Technology 2118) as a loading control. Secondary antibodies used were anti‐mouse immunoglobulin G (IgG) horseradish peroxidase (HRP)–linked antibody and anti‐rabbit IgG HRP‐linked antibody (both from Cell Signaling Technologies).

### Statistical Analysis

Cells were randomly selected for cell speed measurements in all conditions. No pre‐processing of data and exclusion of outliers were performed. All error bars as indicated in figures are mean +/‐ standard error. All sample sizes are indicated in figure captions. All experiments were repeated three or more times with distinct samples that showed similar trend, with the exception of Traction Stress Measurements (*R* = 2) and Actin retrograde flow measurements (*R* = 2). For assessing statistical significance, unpaired Student's *t*‐test was used for all analysis and two experimental groups were compared at a time.

## Conflict of Interest

The authors declare no conflict of interest.

## Author Contributions

*Designed the experiments*: D.M., Y.C., K.K., and S.X.S. *Performed the experiments*: D.M. and K.B. *Analyzed data on actin retrograde flow and performed modeling*: Y.L. and G.Z. *Shared microscope during preliminary data generation*: Y.C. *Wrote the paper*: D.M., K.B., Y.L., K.K., and S.X.S.

## Supporting information

Supporting Information

Supplemental Video 1

Supplemental Video 2

Supplemental Video 3

Supplemental Video 4

Supplemental Video 5

Supplemental Video 6

## Data Availability

The data that support the findings of this study are available from the corresponding author upon reasonable request.
