# Supplementary material for: Extracellular Hydraulic Resistance Enhances Cell Migration
Source: Adv Sci (Weinh). 2022 Aug 28;9(29):2200927. doi: 10.1002/advs.202200927 (PMC9561764; doi:10.1002/advs.202200927)
Supplement: Supplementary file 1 — Supporting Information [file ADVS-9-2200927-s007.pdf]

Supplemental Material

Mathematical Model of Cell Migration Speed in High Viscosity Medium

We use a two-phase, actin-water-osmosis-coupled cell migration model[1,2] to show that the velocity of cell migration increases with the hydraulic resistance and the polarization of the distribution of ion channels, transporters, and pumps. The two phases refer to the cytosol and the F-actin network in the cytoplasm. G-actin is in the cytosol phase. The model also includes ion diffusion and cell surface flux. Active ion fluxes come from actin ion pumps on the cell membrane. For simplicity, we assume that the ions, or solutes, are collectively electro-neutral and the cell migration is approximately steady-state on the time scale being considered.

In the model, we consider a one-dimensional cell segment along the direction of cell migration and use $x \in [0, L]$ to indicate the computational domain established in the moving frame of the cell, where L is the cell length. The conservation of momentum and mass of the cytosol is

$$-\frac{dp}{dx} - \eta\theta_n(v_c - v_n) = 0, \quad \frac{dv_c}{dx} = 0,$$

Where p and v_c are, respectively, the hydraulic pressure and velocity of the cytosol; θ_n and v_n are, respectively, the concentration and velocity of the F-actin network; and η is the coefficient of interfacial friction between the actin-network phase and the cytosol phase due to the velocity difference. The flux boundary condition for cytosol is

$$v_c - v_0 = -J_{\text{water}}^{\text{f}} \quad at\ x = L; \quad v_c - v_0 = J_{\text{water}}^{\text{b}} \quad at\ x = 0,$$

Where J_{water} is the water influx across the cell membrane and the super script 'f' and 'b' indicate quantities evaluated at the front and back end of the cell, respectively. Water flux is driven by the chemical potential difference of water across the cell membrane and its expression is given by

$$J_{\text{water}}^{\text{f(b)}} = -\alpha^{\text{f(b)}}\left[\left(p^{\text{f(b)}} - p_*^{\text{f(b)}}\right) - RT\left(c^{\text{f(b)}} - c_0^{\text{f(b)}}\right)\right],$$

where α is the permeability coefficient of water, c is the concentration of ion, R is the gas constant, and T is the absolute temperature. In the model, we use the subscript '0' to indicate the extracellular environment. Due to the hydraulic resistance, the hydraulic pressure exerting on the outside of the cell, p_*, is different from the hydraulic pressure at infinity, p_0. p_* can be expressed as

$$p_*^{\text{f}} = p_0^{\text{f}} + d_g^{\text{f}}\left(v_0 - J_{\text{water}}^{\text{f}}\right), \quad p_*^{\text{b}} = p_0^{\text{b}} - d_g^{\text{b}}\left(v_0 - J_{\text{water}}^{\text{b}}\right),$$

where d_g is the coefficient of external hydraulic resistance, which proportional to the extracellular medium viscosity[1]. One order of magnitude increase of medium viscosity leads to one order of magnitude increase of the coefficient of external hydraulic resistance. v_0 is the steady-state velocity of the cell.

In this model, we do not explicitly include myosin contraction and we let the pressure in the F-actin network, σ_n, be dominated by the passive pressure due to actin swelling. The constitutive relation for the actin network is modeled as $\sigma_n = k_{\sigma_n}\theta_n$, where k_{σ_n} is a constant. Focal adhesion provides forces to the cell through the actin network. This force can be considered as an effective body force on the network. Therefore, the conservation of momentum of the actin network is written as

$$-\frac{d\sigma_n}{dx} + \eta\theta_n(v_c - v_n) - \eta_{\text{st}}\theta_n v_n = 0,$$

where $\eta_{\rm st}$ is the strength of focal adhesion. In the model, we let actin polymerization occur at the front of the cell and depolymerization occur throughout the cytoplasm. The mass conservation of the F-actin network and G-actin is

$$\frac{d}{dx}(v_n\theta_n) = -\gamma\theta_n \ , \quad \frac{d}{dx}(v_c\theta_c) = D_{\theta_c}\frac{d^2\theta_c}{dx^2} + \gamma\theta_n \ ,$$

where θ_c and D_{θ_c} are, respectively, the concentration and diffusion coefficient of G-actin. γ is a constant rate of actin depolymerization. The boundary condition for F-actin and G-actin is

$$\theta_n(v_0 - v_n) = J_{\rm actin} \ , \quad \theta_c(v_0 - v_c) = -J_{\rm actin}$$

at the front of the cell, where $J_{\rm actin} = J_{\rm actin}^{\rm f}\theta_c/(\theta_{c,c} + \theta_c)$ is the rate of actin polymerization; here $J_{\rm actin}^{\rm f}$ and $\theta_{c,c}$ are two constants. The actin flux is zero at the back of the cell. The total amount of actin is conserved such that $\int_0^L (\theta_n + \theta_c)dx = L\theta_*$, where θ_* is the average concentration of actin.

The diffusion-convection equation for ions is

$$\frac{d}{dx}(v_c c) = D_c\frac{d^2 c}{dx^2} ,$$

where D_c is the diffusion coefficient of ions. The flux at the boundary is a combination of passive, $J_{c,p}$, and active flux, $J_{c,\rm active}$. The passive flux follows the concentration difference of the ion, i.e., $J_{c,p}^{\rm f(b)} = -k_{\rm sol}^{\rm f(b)}(c^{\rm f(b)} - c_0^{\rm f(b)})$, where $k_{\rm sol}$ is the permeability of the passive ion flux. The negative sign comes from the model convention that all fluxes are considered as positive in the inward direction. The active ion flux is prescribed in the model.

The cell experiences a frictional force with the channel wall. We let the friction be proportional to the cell velocity, i.e., $F_f = \xi v_0$, which applies to the negative direction of cell migration. ξ is a friction coefficient. Putting together, the force balance of the entire cell is

$$-(p_0^{\rm f} - p_0^{\rm b}) - (d_g^{\rm f} + d_g^{\rm b})(v_0 - J_{\rm water}^{\rm f}) - \eta_{\rm st}\int_0^L \theta_n v_n dx - F_f = 0.$$

The system is solved by solving all the coupled equations together.

Model Parameters and Results

The polarized ion fluxes are determined by the distribution of membrane ion channels, transporters, and pumps. We use $J_{c,\rm active}^{\rm b}$ and $J_{c,\rm active}^{\rm f}$ to represent the active ion fluxes at the back (trailing edge) and front (leading edge) of the cell, respectively. For polarized cells, $|J_{c,\rm active}^{\rm f}| \neq |J_{c,\rm active}^{\rm b}|$ and we let the ratio $\gamma = |J_{c,\rm active}^{\rm f}/J_{c,\rm active}^{\rm b}|$ reflect the level of ion pump polarization.

The list of parameters used in the model can be found in Tab. 1.

Tab. 1. Default parameters used in the model.

**Parameters**	**Description**	**Values**	**Sources**

R (J/mol/K)	Ideal gas constant	8.31	Constant
T (K)	Absolute temperature	310	Physiological condition
L (μm)	Cell length	50	From experiment
η (Pa s/ μm^2/mM)	Interfacial drag coefficient	10^{-2}	Dembo & Harlow (1986)[3]
η_{st} (Pa s/ μm^2/mM)	Focal adhesion strength coefficient	10	Based on Li et al. (2019)[2]
k_{σ_n} (Pa/mM)	Passive F-actin stress coefficient	100	Estimated
k_{ad} (Pa s/μm)	Coefficient of adhesive force	10	Estimated
d_g (Pa s/μm)	Coefficient of hydraulic resistance	1	Based on Li et al. (2019)[2]
$J_{\mathrm{actin}}^{\mathrm{f}}$ (nm mM/s)	Coefficient of actin polymerization	1	Based on Li et al. (2019)[2]
$\theta_{c,c}$ (μM)	Critical G-actin concentration	0.2	Pollard et al. (2000)[4]
γ (1/s)	Constant rate of actin depolymerization	5 x 10^{-4}	Based on Kuhn (2007)[5]
θ_* (mM)	Average actin concentration	300	Satcher & Dewey (1996)[6], Pollard et al. (2000)[4]
D_c (μm^2/s)	Diffusion coefficient of ion	100	Stroka et al. (2014)[7]
D_{θ_c} (μm^2/s)	Diffusion coefficient of G-actin	10	Estimated
$k_{\mathrm{sol}}^{\mathrm{f(b)}}$ (μm/s)	Passive ion channel permeability	50	Li et al. (2019)[2]
$J_{c,\mathrm{active}}^{\mathrm{b}}$ (μm mM/s)	Active ion flux at the back	16.4	Based on Li et al. (2019)[2]
$J_{c,\mathrm{active}}^{\mathrm{f}}$ (μm mM/s)	Active ion flux at the front	32.8	Based on Li et al. (2019)[2]
$\alpha^{\mathrm{f(b)}}$ (μm/Pa/s)	Water permeability	10^{-4}	Li et al. (2019)[2]
$p_0^{\mathrm{f(b)}}$ (Pa)	Hydraulic pressure at infinity	0	Free constant
$c_0^{\mathrm{f(b)}}$ (mM)	Extracellular ion concentration	340	Physiological range

In the experiment, we first observed cell velocity increase in high-viscosity media (Fig 1B) while the actin expression and focal adhesion strength remain unchanged (Fig. 2). High-viscosity media create a high coefficient of hydraulic resistance, d_g, for the cell[1,2]. The hydraulic resistance-induced cell velocity increase is predicted by the model (Fig. 6A, left panel). A three-order of magnitude increase in the coefficient of hydraulic resistance can increase the cell velocity by 20 μm/h. The result indicates that the water-driven component of cell migration is more affected by the hydraulic resistance.

The water-driven component comes from polarized distribution of ion transporter and pumps that creates osmosis gradient and directional water flux across the cell. The model predicts that the water-driven component of cell velocity increases with the ion pump polarization (Fig. 6B, right panel). Inhibition of ion transporters and pumps will remove the polarity and leads to cell velocity reduction. Indeed, we have observed from experiments that inhibition of NaK or dual inhibition of NHE and NKCC leads to cell velocity decrease (Fig.3).

More interestingly, inhibition of NaK has a more significant effect on cell velocity when cells are in high-viscosity medium (Fig. 3E). This is because in high viscosity, i.e, high hydraulic resistance, the contribution from water flux to cell velocity is more prominent (Fig. 6B)). A reduction of water flux due to NaK inhibition is thus predicted to have higher impact on cell velocity.

References

1. Li, Y. & Sun, S. X. Transition from Actin-Driven to Water-Driven Cell Migration Depends on External Hydraulic Resistance. *Biophys. J.* **114**, 2965–2973 (2018).
2. Li, Y., Yao, L., Mori, Y. & Sun, S. X. On the energy efficiency of cell migration in diverse physical environments. *Proc. Natl. Acad. Sci.* **116**, 23894–23900 (2019).
3. Dembo, M. & Harlow, F. Cell motion, contractile networks, and the physics of interpenetrating reactive flow. *Biophys. J.* **50**, 109–121 (1986).
4. Pollard, T. D., Blanchoin, L. & Mullins, R. D. Molecular Mechanisms Controlling Actin Filament Dynamics in Nonmuscle Cells. *Annu. Rev. Biophys. Biomol. Struct.* **29**, 545–576 (2000).
5. Kuhn, J. R. & Pollard, T. D. Single Molecule Kinetic Analysis of Actin Filament Capping. *J. Biol. Chem.* **282**, 28014–28024 (2007).
6. Satcher, R. L. & Dewey, C. F. Theoretical estimates of mechanical properties of the endothelial cell cytoskeleton. *Biophys. J.* **71**, 109–118 (1996).
7. Stroka, K. M. *et al.* Water Permeation Drives Tumor Cell Migration in Confined Microenvironments. *Cell* **157**, 611–623 (2014).

Supplementary Figure Captions:

Figure S1- Cell migration speed and total distance travelled increase with viscosity for multiple cell lines and investigation of persistence as a function of viscosity (A) Schematic representation of cell persistence. Higher persistence is characterized by a lower angle θ. **(B), (C)** Total distance travelled and persistence of MDA-MB 231 cells in DMEM (N=253) and 1%MC (N=213), respectively. **(D)** Speed of 3T3 cells in DMEM (N=247) and 1%MC (N=176). **(E), (F)** Total distance travelled and persistence of 3T3 cells in DMEM (N=247) and 1%MC (N=176). **(G)** Speed of HT1080 cells in DMEM (N=165) and 1%MC (N=119). **(H), (I)** Total distance travelled and persistence of HT1080 cells in DMEM (N=165) and 1%MC (N=119). **(J)** Speed of Sum159 cells in DMEM (N=221) and 1%MC (N=176). **(K), (L)** Total distance travelled and persistence of Sum159 cells in DMEM (N=221) and 1%MC (N=176). *$p<0.05$, **$p<0.005$ and ***$p<0.0005$ across all plots. Error bars represent standard deviation of the data.

Figure S2- Traction stress does not change in high viscosity media (A) Comparisons of traction stress obtained from traction force microscopy for cells in DMEM (N=5) and 1%MC (N=10), *$p<0.05$, **$p<0.005$ and ***$p<0.0005$. Error bars represent standard deviation of the data.

Figure S3- Total NKCC1, NHE1, NaK do not change in high viscosity media, while the ion-channel content at cell leading edge is more in normal viscosity media, total F-Actin does not change upon inhibition of ion-channel activity, NKCC1 per unit F-Actin reduces in NKCC1 Knockdown cells and AQP5 knock-down inhibits cell speed in high viscosity media (A) Total immunofluorescence intensity of NKCC1 (DMEM N=157; 1%MC N=124) for cells in low and high viscosity media. **(B)** Total immunofluorescence intensity of NHE1 in cells in low and high viscosity media (DMEM N=161; 1%MC N=106). **(C)** Total immunofluorescence intensity of NaK in cells in low and high viscosity media (DMEM N=121; 1%MC N=96). **(D)** Ion-channel at the cell leading edge is more in case of low viscosity media compared to high viscosity media. Leading Edge intensity comparison of NKCC1 (DMEM N=108; 1%MC N=124) **(E)** Leading Edge intensity comparison of NHE1 (DMEM N=107; 1%MC N=82) **(F)** Leading Edge intensity comparison of NaK (DMEM N=124; 1%MC N=119). **(G)** Comparisons of total F-actin in cells after inhibition of ion-channel activity, either through dual- inhibition of NKCC1 and NHE1 via 30μM Bumetanide and 20μM EipA, or, through inhibition of NaK via 50μM Ouabain [1%MC DMSO Ctrl (N=75), 1%MC Dual inhibition (N=61), 1%MC 50μM Ouabain (N=51)]. **(H)** NKCC1 to F-actin ratio in control (Scr Ctrl N=240) and in NKCC1 knockdowns (S1 N=185; S2 N=238; S3 N=125). AQP5 KD inhibits cell speed in high viscosity media **(I)** DMEM Scr Ctrl (N=120), 1%MC Scr Ctrl (N=105), DMEM AQP5 KD (N=169), 1%MC AQP5 KD (N=148). **(J)** Total distance travelled for NKCC1 knockdown cells with 20μM EIPA (DMEM Scr Ctrl N=49; 1%MC Scr Ctrl N=63; DMEM S1 N=61; 1%MC S1 N=68; DMEM S2 N=39; 1%MC S2 N=44; DMEM S3 N=47; 1%MC S3 N=51). **(K)** Cell Area change for NKCC1 knockdown cells with 20μM EIPA (DMEM Scr Ctrl N=14; 1%MC Scr Ctrl N=17; DMEM S1 N=13; 1%MC S1 N=15; DMEM S2 N=22; 1%MC S2 N=20; DMEM S3 N=17; 1%MC S3 N=22). **(L)** Cell Area change upon dual- inhibition of NKCC1 and NHE1 via 30μM Bumetanide and 20μM EipA [DMEM DMSO (N=82), 1%MC DMSO (N=80), DMEM Dual inhibition N=30), 1%MC Dual inhibition (N=41)]. **(M)** Cell area change upon treatment with 50μM Ouabain [DMEM DMSO (N=82), 1%MC DMSO (N=80), DMEM 50μM Ouabain (N=31), 1%MC 50μM Ouabain (N=42)]. **(N)** Total distance travelled for NKCC1 and NHE1 double knockdown (dKD) (DMEM Scr Ctrl N=77; 1%MC Scr Ctrl N=77; DMEM dKD1 N=86; 1%MC dKD1 N=85; DMEM dKD2 N=81; 1%MC dKD2 N=80). **(O)** Cell Area change for NKCC1 and NHE1 double knockdown (dKD) (DMEM Scr Ctrl N=90; 1%MC Scr Ctrl N=58; DMEM dKD1 N=83; 1%MC dKD1 N=40; DMEM dKD2 N=76; 1%MC dKD2 N=61). **(P)** Western blot of the knockdown efficiency of shRNA mediated dual knock down of NHE1 and NKCC1. Image representative of 2 independent protein extractions. **(Q)** NKCC1 and NKCC1,

F-Actin normalized polarized comparison between DMEM (N=29) and 1%MC (N=36) **(R)** NHE1 and NHE1, F-Actin normalized polarization comparison between DMEM (N=17) and 1%MC (N=20). **(S)** NaK and NaK, F-Actin normalized polarization comparison between DMEM (N=31) and 1%MC (N=23). *p<0.05, **p<0.005 and ***p<0.0005 across all plots. Error bars represent standard deviation of the data.

Figure S4- Total NKCC1, NHE1, NaK and F-Actin upon perturbation of vesicle trafficking. (A) Total immunofluorescence intensity of NKCC1 (1%MC DMSO Ctrl N=59; 1%MC 2.5nM LatA N=88; 1%MC 50μM MyoVinI N=64; 1%MC 2.5μl DMSO Ctrl N=53; 1%MC 250μM CID 1067700 N=45). **(B)** Total immunofluorescence intensity of NHE1 (1%MC DMSO Ctrl N=57; 1%MC 2.5nM LatA N=66; 1%MC 50μM MyoVinI N=55; 1%MC 2.5μl DMSO Ctrl N=81; 1%MC 250μM CID 1067700 N=54). **(C)** Total immunofluorescence intensity of NaK (1%MC DMSO Ctrl N=108; 1%MC 2.5nM LatA N=103; 1%MC 50μM MyoVinI N=62; 1%MC 2.5μl DMSO Ctrl N=52; 1%MC 250μM CID 1067700 N=59). **(D)** Total immunofluorescence intensity of F-actin (1%MC DMSO Ctrl N=224; 1%MC 2.5nM LatA N=257; 1%MC 50μM MyoVinI N=181; 1%MC 2.5μl DMSO Ctrl N=186; 1%MC 250μM CID 1067700 N=158). **(E)** Cell area change upon Latrunculin A treatment (DMEM DMSO Ctrl N=82; 1%MC DMSO Ctrl N=80; DMEM 2.5nM LatA N=44; 1%MC 2.5nM LatA N=31); Cell area change upon Myosin V inhibition via MyoVinI (DMEM DMSO Ctrl N=82; 1%MC DMSO Ctrl N=80; DMEM 50μM MyoVinI N=33; 1%MC 50μM MyoVinI N= 35); Cell area change upon Rab7 inhibition via CID 1067700 (DMEM DMSO Ctrl N=28; 1%MC DMSO Ctrl N=22; DMEM 250μM CID 1067700 N=61; 1%MC 250μM CID 1067700 N= 54). *p<0.05, **p<0.005 and ***p<0.0005 across all plots. Error bars represent standard deviation of the data.

Figure S5- Quantification of Ca^{2+} dynamics of cells in normal and high viscosity media and Ca^{2+} chelation by BAPTA-AM does not cause a change in total ion-channel/ F-Actin. (A), (B) Mean relative peak and maximum relative peak power (see calcium imaging section of Materials and Methods) of cells in 3 different conditions: DMEM (immediately after addition) (N=58), 1%MC (immediately after addition) (N=61) and 1%MC (long term >3hrs incubation) (N=39). Intracellular Ca^{2+} levels are higher after addition of high viscosity media (1%MC). **(C), (D)** Intracellular Ca^{2+} levels of cells immediately after the addition of DMEM (Blue) and 1%MC (Red) over a short duration (~10min) (DMEM N=16; 1%MC N=29). **(E), (F)** Intracellular Ca^{2+} levels of cells immediately after the addition of DMEM (Blue) and 1%MC (Red) over a long duration (~2.5hrs) (DMEM N=20; 1%MC N=34). **(G)** Representative immunofluorescence images of NKCC1, NHE1, NaK and F-actin of cells in DMSO Ctrl and with the treatment of 10μM BAPTA-AM. Total ion-channel and F-actin levels do not change under BAPTA-AM treatment. **(H)** Total immunofluorescence intensity of NKCC1 in 1%MC DMSO Ctrl (N=93) and in 1%MC 10μM BAPTA-AM (N=62). **(I)** Total immunofluorescence intensity of NHE1 in 1%MC DMSO Ctrl (N=87) and in 1%MC 10μM BAPTA-AM (N=70). **(J)** Total immunofluorescence intensity of NaK in 1%MC DMSO Ctrl (N=69) and in 1%MC 10μM BAPTA-AM (N=66). **(K)** Total F-actin intensity in 1%MC DMSO Ctrl (N=249) and in 1%MC 10μM BAPTA-AM (N=198). **(L)** Total distance travelled upon treatment with 2-APB and CAI (DMEM DMSO Ctrl N=99; 1%MC DMSO Ctrl N=102; DMEM 200μM 2-APB + 40μM CAI N=116; 1%MC 200μM 2-APB + 40μM CAI N=118). **(M)** Cell area change upon Ca^{2+} chelation via BAPTA-AM (DMEM DMSO Ctrl N=82; 1%MC DMSO Ctrl N=80; DMEM 10μM BAPTA-AM N=25; 1%MC 10μM BAPTA-AM N=37). **(N)** Cell area change upon calpain inhibition via calpain inhibitor I (DMEM DMSO Ctrl N=82; 1%MC DMSO Ctrl N=80; DMEM 2.5μM Calpain inhibitor I N=26; 1%MC 2.5μM Calpain inhibitor I N=28). **(O)** Cell area change upon treatment with 2-APB and CAI (DMEM DMSO Ctrl N=67; 1%MC DMSO Ctrl N=68; DMEM 200μM 2-APB + 40μM CAI N=65; 1%MC 200μM 2-APB + 40μM CAI N=70). *p<0.05, **p<0.005 and ***p<0.0005 across all plots. Error bars represent standard deviation of the data.

Figure S6- Inhibition of water permeation affects cell migration speed in 3D. 2D migration speed increase in high viscosity media is less with collagen coating owing to the fact that motility is more actin driven in former. (A) Cell migration speed in 3D collagen gels under various drug treatments (DMSO Ctrl N=22; DMEM 30μM Bumetanide N=42; DMEM 20μM EIPA N=59; DMEM 50μM Ouabain N=59; DMEM 50μM MyoVinI N=80; DMEM 2.5μl DMSO Ctrl N=49; DMEM 250μM CID 1067700 N=73). **(B)** Cell speed in DMEM (N=162) and 1%MC (N=145) with 20μg/ml collagen coating on glass substrates. Both mean and total F-actin levels increase in cells with collagen coating. **(C), (D)** Comparisons of mean and total F-actin in cells without collagen coating [DMEM (N=300) and 1%MC (N=250)] and with 20μg/ml collagen coating [DMEM (N=51) and 1%MC (N=58)]. **(E)** Confocal measurement of fluorescent Dextran in cells over time in high viscosity media. Fluorescent images of cells incubated in 1%MC with 0.1mg/ml of 150kDa FITC Dextran at different time points. No significant intracellular Dextran intensity change is observed during the time period of cell speed measurement, indicating endocytosis is not significant in 1%MC (N=7 cells)**.** *p<0.05, **p<0.005 and ***p<0.0005 across all plots. Error bars represent standard deviation of the data.

Supplementary Movies

SM 1: Video demonstrating cell speed increase in high viscosity media (1%MC) compared to normal media (DMEM). DMEM (Left), 1%MC (Right). Acquisition rate is 10 min per frame.

SM 2: Video demonstrating retrograde flow speed reduction in high viscosity media (1%MC) compared to normal media (DMEM). DMEM (Left), 1%MC (Right). Acquisition rate is 3 sec per frame.

SM 3A & 3B: Video demonstrating more speed reduction in high viscosity media (1%MC) compared to normal media (DMEM) upon treatment with 25μM Ouabain. SM 3A- DMEM DMSO (Left), 1%MC DMSO (Right). SM 3B- DMEM 25μM Ouabain (Left), 1%MC 25μM Ouabain (Right). Acquisition rate is 10min per frame.

SM 4A & 4B: Video demonstrating more speed reduction in high viscosity media (1%MC) compared to normal media (DMEM) upon treatment with 250μM Rab7 inhibitor. SM 4A- DMEM DMSO (Left), 1%MC DMSO (Right). SM 4B- DMEM 250μM Rab7 inhibitor (Left), 1%MC 250μM Rab7 inhibitor (Right). Acquisition rate is 10min per frame.

SM 5: Video demonstrating more speed reduction in high viscosity media (1%MC) compared to normal media (DMEM) upon treatment with 200μM 2-APB + 40μM CAI. SM 5A- DMEM DMSO (Left), 1%MC DMSO (Right). SM 5B- DMEM 200μM 2-APB + 40μM CAI (Left), 1%MC 200μM 2-APB + 40μM CAI (Right). Acquisition rate is 10min per frame.

SM 6: Video demonstrating high fluctuations in Ca^{2+} signals when cells come in contact with high viscosity media (1%MC) compared to normal media (DMEM) or long-term incubation in high viscosity media (1%MC). Cells, immediately, when they come in contact with DMEM (Left), cells, immediately, when they come in contact with 1%MC (Middle), cells, when they are incubated in high viscosity media 1%MC for >3hrs (Right). Acquisition rate is 1 sec per frame.

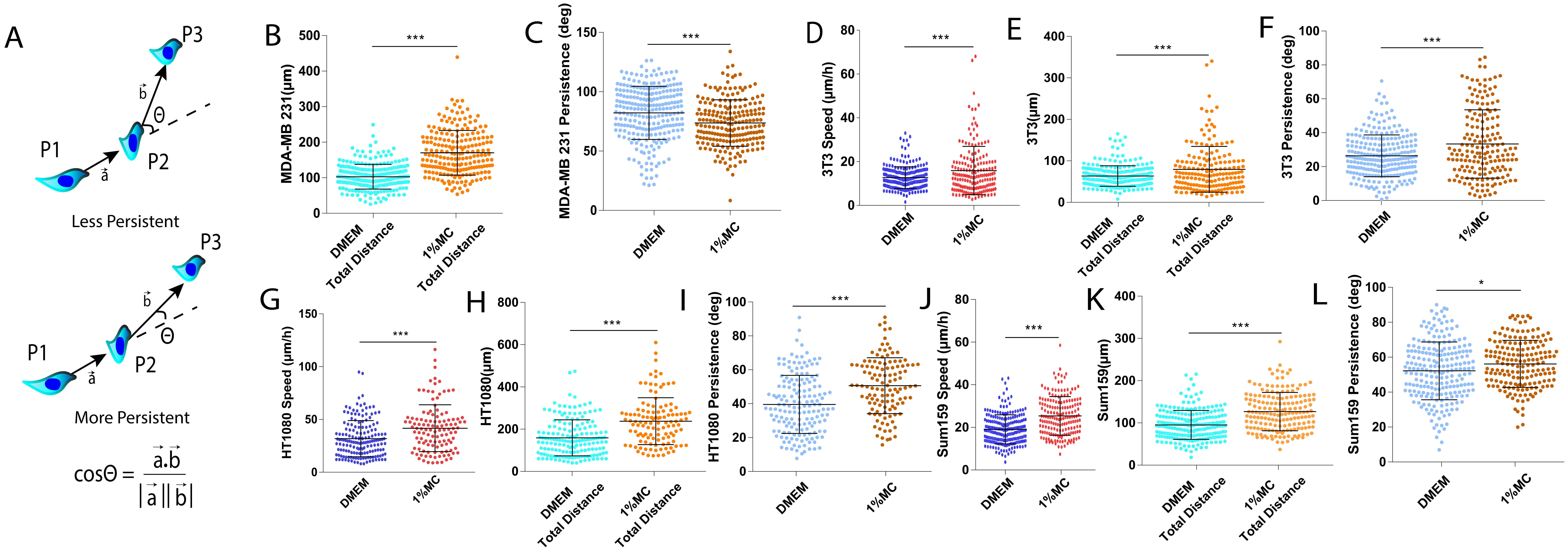
A
P1
P2
P3
Θ
Less Persistent
More Persistent
cosΘ = a.b / |a||b|
B
MDA-MB 231(μm)
DMEM Total Distance
1%MC Total Distance
***
C
MDA-MB 231 Persistence (deg)
DMEM
1%MC
D
3T3 Speed (μm/h)
E
3T3(μm)
F
3T3 Persistence (deg)
G
HT1080 Speed (μm/h)
H
HT1080(μm)
I
HT1080 Persistence (deg)
J
Sum159 Speed (μm/h)
K
Sum159(μm)
L
Sum159 Persistence (deg)
*

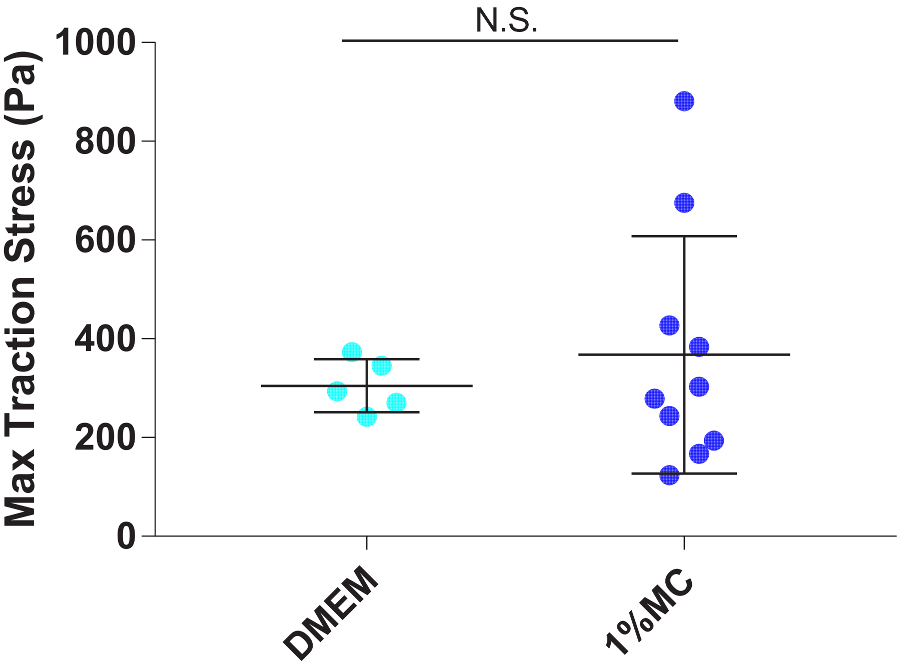
N.S.
Max Traction Stress (Pa)
1000
800
600
400
200
0
DMEM
1%MC

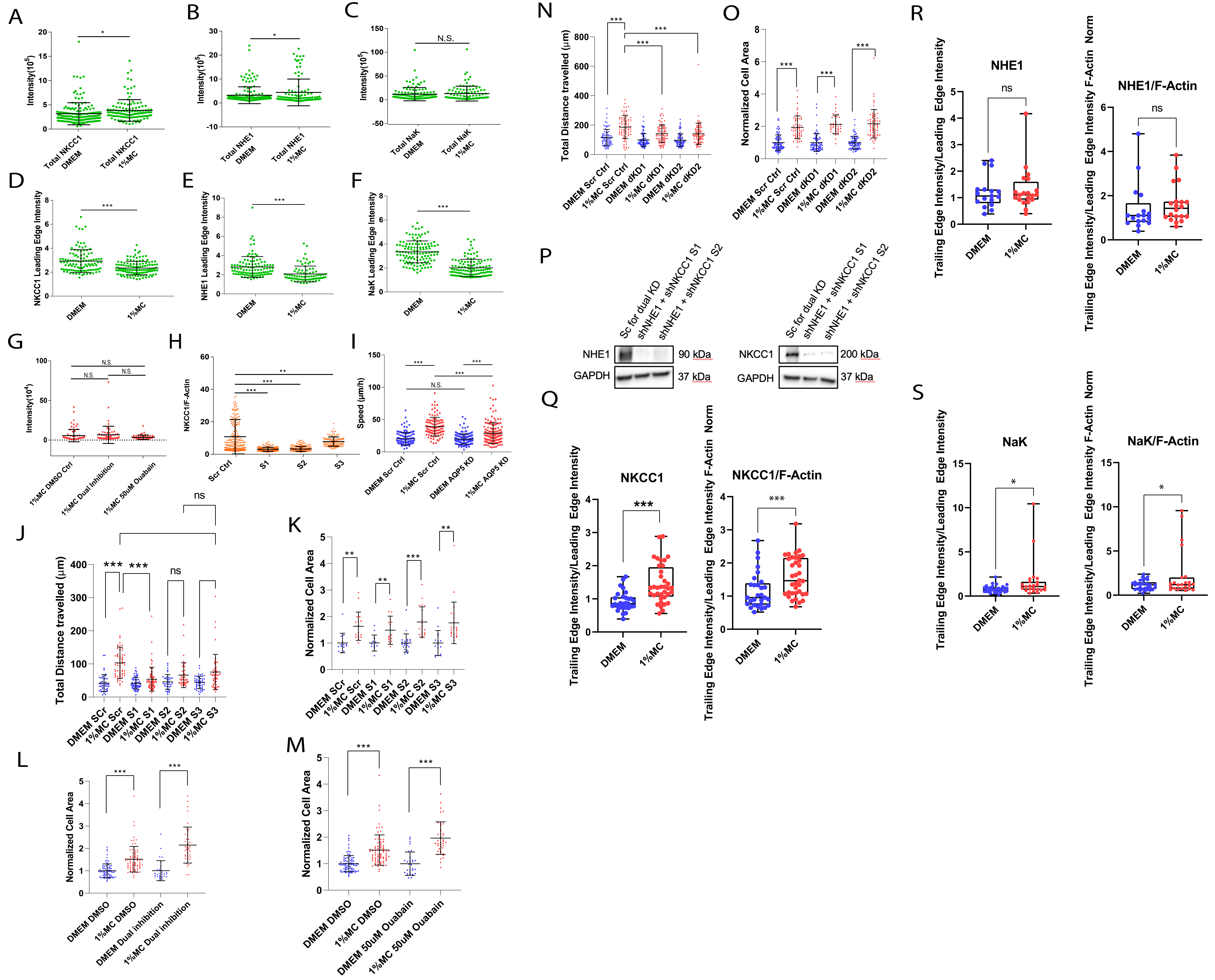
A
Intensity(10^5)
Total NKCC1 DMEM
Total NKCC1 1%MC
B
Intensity(10^5)
Total NHE1 DMEM
Total NHE1 1%MC
C
N.S.
Intensity(10^5)
Total NaK DMEM
Total NaK 1%MC
D
NKCC1 Leading Edge Intensity
DMEM
1%MC
E
NHE1 Leading Edge Intensity
F
NaK Leading Edge Intensity
G
N.S.
Intensity(10^4)
1%MC DMSO Ctrl
1%MC Dual Inhibition
1%MC 50uM Ouabain
H
NKCC1/F-Actin
Scr Ctrl
S1
S2
S3
I
Speed (µm/h)
DMEM Scr Ctrl
1%MC Scr Ctrl
DMEM AQP5 KD
1%MC AQP5 KD
J
ns
Total Distance travelled (µm)
DMEM SCr
1%MC Scr
DMEM S1
1%MC S1
DMEM S2
1%MC S2
DMEM S3
1%MC S3
K
Normalized Cell Area
DMEM SCr
1%MC Scr
DMEM S1
1%MC S1
DMEM S2
1%MC S2
DMEM S3
1%MC S3
L
Normalized Cell Area
DMEM DMSO
1%MC DMSO
DMEM Dual inhibition
1%MC Dual inhibition
M
Normalized Cell Area
DMEM DMSO
1%MC DMSO
DMEM 50uM Ouabain
1%MC 50uM Ouabain
N
Total Distance travelled (µm)
DMEM Scr Ctrl
1%MC Scr Ctrl
DMEM dKD1
1%MC dKD1
DMEM dKD2
1%MC dKD2
O
Normalized Cell Area
DMEM Scr Ctrl
1%MC Scr Ctrl
DMEM dKD1
1%MC dKD1
DMEM dKD2
1%MC dKD2
P
Sc for dual KD
shNHE1 + shNKCC1 S1
shNHE1 + shNKCC1 S2
NHE1
90 kDa
GAPDH
37 kDa
NKCC1
200 kDa
GAPDH
37 kDa
Q
NKCC1
Trailing Edge Intensity/Leading Edge Intensity
NKCC1/F-Actin
Trailing Edge Intensity/Leading Edge Intensity F-Actin Norm
DMEM
1%MC
R
NHE1
ns
Trailing Edge Intensity/Leading Edge Intensity
NHE1/F-Actin
Trailing Edge Intensity/Leading Edge Intensity F-Actin Norm
DMEM
1%MC
S
NaK
Trailing Edge Intensity/Leading Edge Intensity
NaK/F-Actin
Trailing Edge Intensity/Leading Edge Intensity F-Actin Norm
DMEM
1%MC

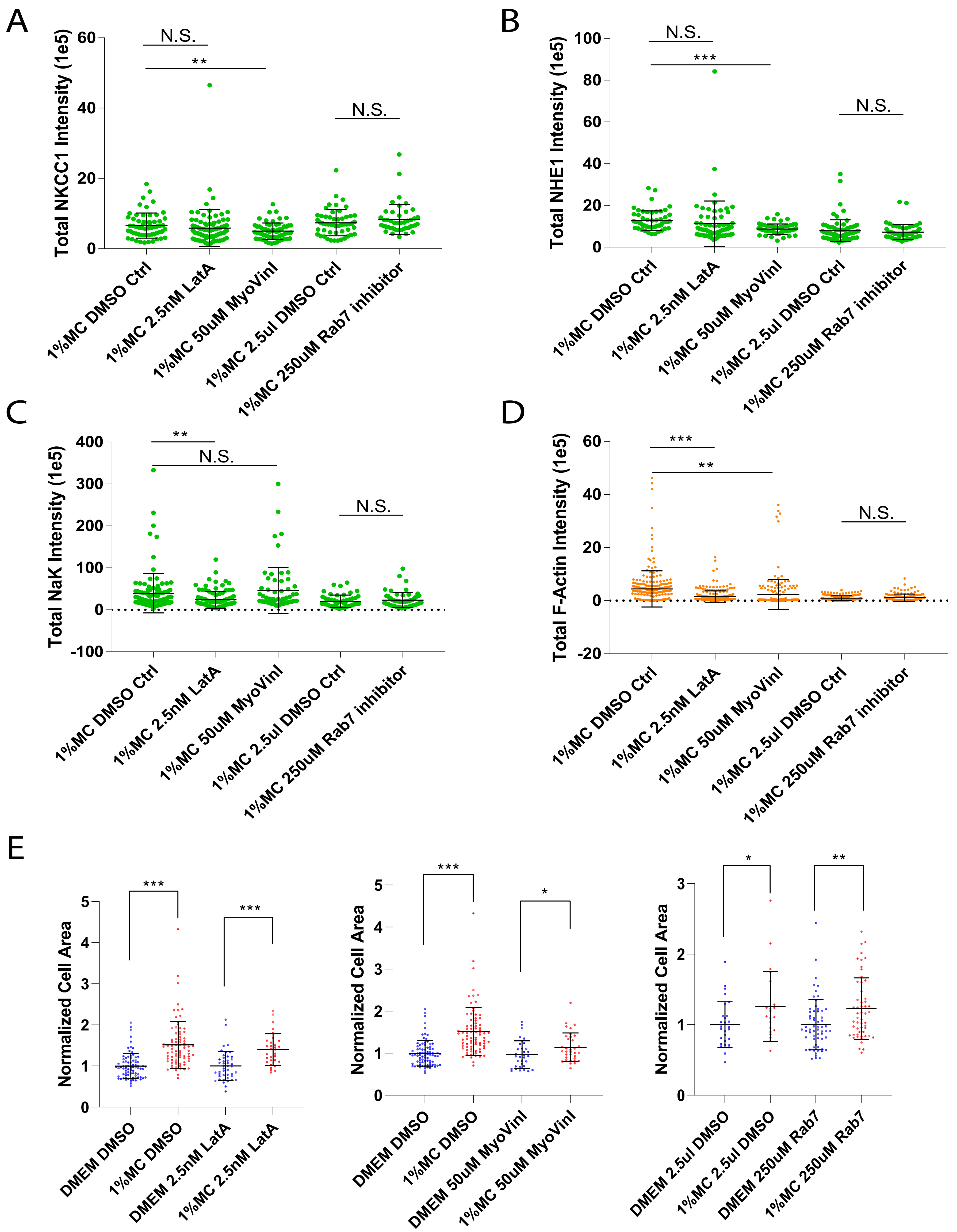
A
Total NKCC1 Intensity (1e5)
N.S.
**
N.S.
1%MC DMSO Ctrl
1%MC 2.5nM LatA
1%MC 50uM MyoVinI
1%MC 2.5ul DMSO Ctrl
1%MC 250uM Rab7 inhibitor
B
Total NHE1 Intensity (1e5)
N.S.
***
N.S.
C
Total NaK Intensity (1e5)
**
N.S.
N.S.
D
Total F-Actin Intensity (1e5)
***
**
N.S.
E
Normalized Cell Area
DMEM DMSO
1%MC DMSO
DMEM 2.5nM LatA
1%MC 2.5nM LatA
DMEM 50uM MyoVinI
1%MC 50uM MyoVinI
DMEM 2.5ul DMSO
1%MC 2.5ul DMSO
DMEM 250uM Rab7
1%MC 250uM Rab7

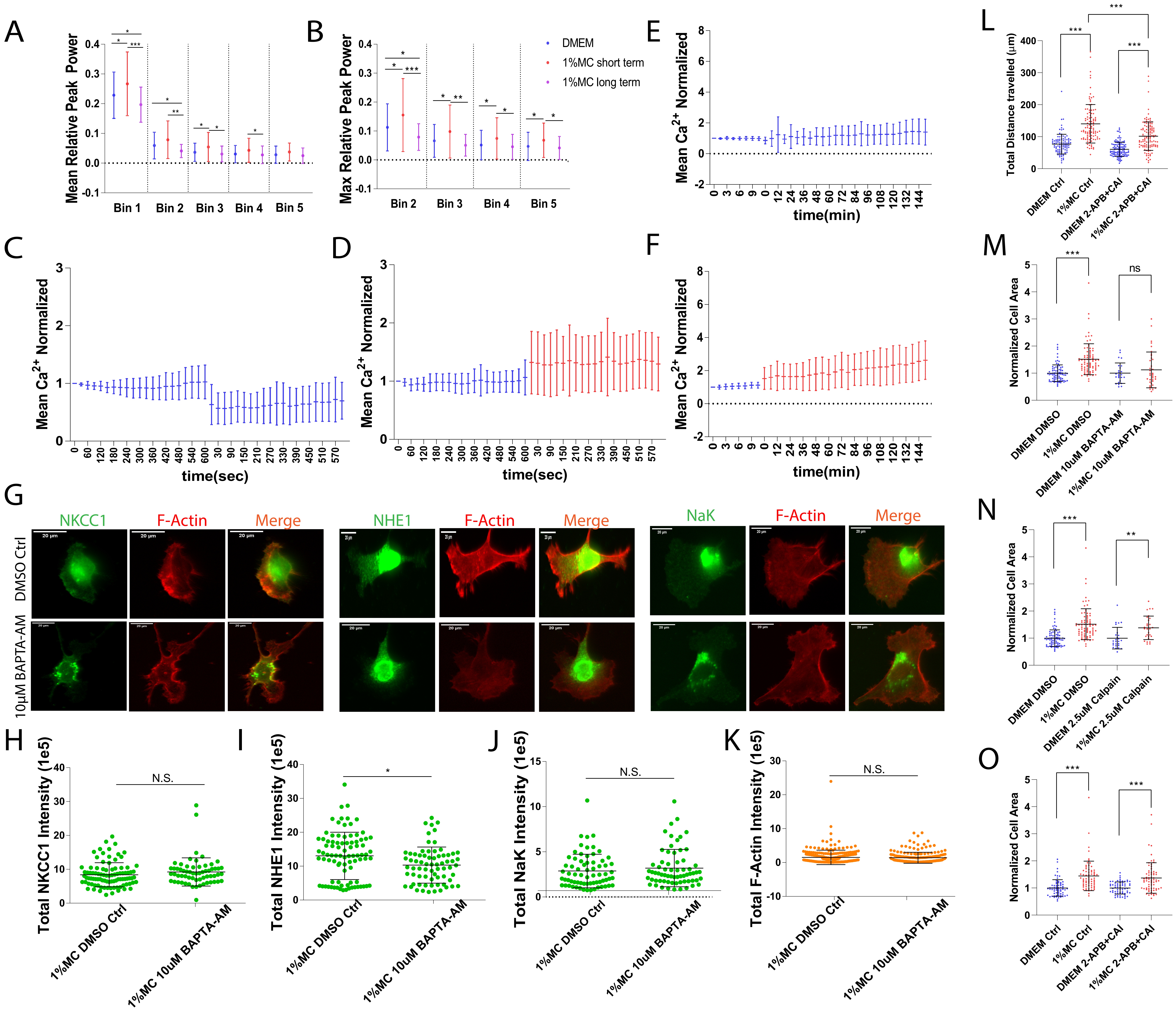
A
Mean Relative Peak Power
Bin 1
Bin 2
Bin 3
Bin 4
Bin 5
B
Max Relative Peak Power
DMEM
1%MC short term
1%MC long term
E
Mean Ca2+ Normalized
time(min)
L
Total Distance travelled (μm)
DMEM Ctrl
1%MC Ctrl
DMEM 2-APB+CAI
1%MC 2-APB+CAI
C
D
time(sec)
F
M
Normalized Cell Area
DMEM DMSO
1%MC DMSO
DMEM 10uM BAPTA-AM
1%MC 10uM BAPTA-AM
ns
G
NKCC1
F-Actin
Merge
NHE1
NaK
DMSO Ctrl
10μM BAPTA-AM
20 μm
N
DMEM 2.5uM Calpain
1%MC 2.5uM Calpain
H
Total NKCC1 Intensity (1e5)
N.S.
1%MC DMSO Ctrl
1%MC 10uM BAPTA-AM
I
Total NHE1 Intensity (1e5)
J
Total NaK Intensity (1e5)
K
Total F-Actin Intensity (1e5)
O

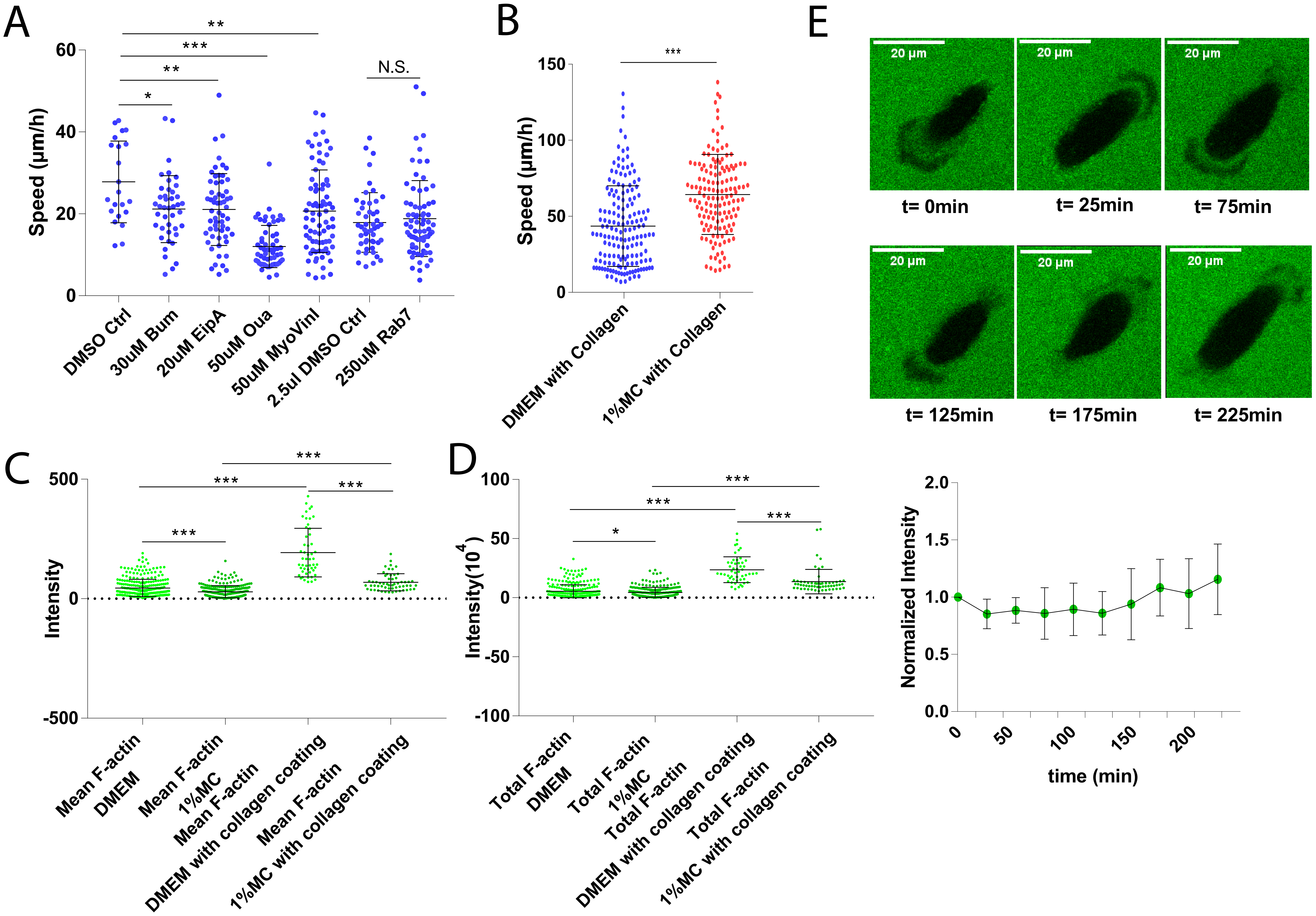
A
Speed (μm/h)
60
40
20
0
**
***
**
*
N.S.
DMSO Ctrl
30uM Bum
20uM EipA
50uM Oua
50uM MyoVinl
2.5ul DMSO Ctrl
250uM Rab7
B
Speed (μm/h)
150
100
50
0
***
DMEM with Collagen
1%MC with Collagen
E
20 μm
t= 0min
t= 25min
t= 75min
t= 125min
t= 175min
t= 225min
C
Intensity
500
0
-500
***
Mean F-actin DMEM
Mean F-actin 1%MC
Mean F-actin DMEM with collagen coating
Mean F-actin 1%MC with collagen coating
D
Intensity(10^4)
100
50
0
-50
-100
*
Total F-actin DMEM
Total F-actin 1%MC
Total F-actin DMEM with collagen coating
Total F-actin 1%MC with collagen coating
Normalized Intensity
2.0
1.5
1.0
0.5
0.0
0
50
100
150
200
time (min)
